# Hyaluronic Acid-Functionalized Nanomicelles Enhance SAHA Efficacy in 3D Endometrial Cancer Models

**DOI:** 10.3390/cancers13164032

**Published:** 2021-08-10

**Authors:** Kadie Edwards, Seydou Yao, Simone Pisano, Veronica Feltracco, Katja Brusehafer, Sumanta Samanta, Oommen P. Oommen, S. Andrea Gazze, Roberta Paravati, Holly Maddison, Chao Li, Deyarina Gonzalez, R. Steven Conlan, Lewis Francis

**Affiliations:** 1 Reproductive Biology and Gynaecological Oncology Group, Swansea University Medical School, Singleton Park, Swansea SA2 8PP, UK; 832659@swansea.ac.uk (K.E.); S.N.Yao@swansea.ac.uk (S.Y.); 966634@swansea.ac.uk (S.P.); veronica.feltra@gmail.com (V.F.); 942084@swansea.ac.uk (K.B.); S.A.Gazze@swansea.ac.uk (S.A.G.); r.paravati@swansea.ac.uk (R.P.); 794300@swansea.ac.uk (H.M.); D.Gonzalez@swansea.ac.uk (D.G.); R.S.Conlan@swansea.ac.uk (R.S.C.); 2 Bioengineering and Nanomedicine Lab, Faculty of Medicine and Health Technology, Tampere University and BioMediTech Institute, 33720 Tampere, Finland; sumanta.samanta@tuni.fi (S.S.); oommen.oommen@tuni.fi (O.P.O.); 3 Suzhou School of Nano-Science and Nano-Engineering, Xi’an Jiaotong University, Suzhou Industrial Park, Suzhou 215123, China; cli12@xjtu.edu.cn

**Keywords:** polymeric nanoparticles, hyaluronic acid, epigenetics, SAHA, nanomedicines, gynecological oncology, F127 micelles, targeted therapeutics, 3D models

## Abstract

**Simple Summary:**

One of the major limitations to cancer therapies are the side effects caused by the drug interacting with any tissue in the body. There is often a balance between patient health and effectively treating the disease. To by-pass this balancing act nanoparticles are being used to deliver therapeutics straight to the tumors, acting as “Trojan Horses”. Endometrial cancers are known to have more of the cell surface protein CD44 than healthy tissues. Here, to efficiently target endometrial cancer, hyaluronic acid, which naturally binds to the CD44 protein was attached to the surface of nanoparticles and tested on microtissues or spheroids to better model a tumor and understand drug delivery performance. We show that our hyaluronic acid-nanoparticle formulations improve drug effects and interact with the cancer cells more than without this targeting agent.

**Abstract:**

Histone Deacetylase (HDAC) enzymes are upregulated in cancer leading to the development of HDAC inhibiting compounds, several of which are currently in clinical trials. Side effects associated with toxicity and non-specific targeting indicate the need for efficient drug delivery approaches and tumor specific targeting to enhance HDAC efficacy in solid tumor cancers. SAHA encapsulation within F127 micelles functionalized with a surface hyaluronic acid moiety, was developed to target endometrial cancer cells expressing elevated levels of CD44. In vitro viability and morphology analyses was conducted in both 2D and 3D models to assess the translational potential of this approach. Encapsulation enhanced SAHA delivery and activity, demonstrating increased cytotoxic efficacy in 2D and 3D endometrial cancer models. High-content imaging showed improved nanoparticle internalization in 2D and CD44 enhanced penetration in 3D models. In addition, the nano-delivery system enhanced spheroid penetration resulting in cell growth suppression, p21 associated cell cycle arrest, as well as overcoming the formation of an EMT associated phenotype observed in free drug treated type II endometrial cancer cells. This study demonstrates that targeted nanoparticle delivery of SAHA could provide the basis for improving its efficacy in endometrial cancer. Using 3D models for endometrial cancer allows the elucidation of nanoparticle performance and CD44 targeting, likely through penetration and retention within the tumor model.

## 1. Introduction

Endometrial cancer is the sixth most common cancer in women and the 15th most commonly occurring cancer worldwide, with over 380,000 new cases reported in 2018 [1,2,3]. The disease is most prevalent in post-menopausal women, with a dramatic 13% increase in the incidence reported in the UK between 2007–2017. Alarmingly this rate is exacerbated in younger women aged 25 to 45 years, [2,4] and has been linked to genetic factors, obesity and poor diet, with an elevated risk in patients suffering from polycystic ovary syndrome [5,6].

Endometrial cancer can broadly be divided to type I or II, with disease progression further defined in six stages, according to the Rotterdam criteria [7]. Type I endometrioid adenocarcinoma, representing 80% of all cases [8], is a low-grade, hormone receptor positive disease with a good prognostic outcome if diagnosed early [6]. Many stage I cancers can be cured with surgery alone, although more advanced stages require adjuvant radiotherapy or chemotherapies such as paclitaxel and ifosfamide to avoid reoccurrence [9]. In contrast, type II endometrial carcinomas are estrogen-independent, often with high-grade serous or clear cell histology and poor outcomes, and account for 40% of all endometrial cancer associated mortality [4,10]. Advanced stage endometrial cancers become metastatic, to the point where surgery is not the most appropriate treatment option. Chemotherapy then becomes preferred, with cisplatin, carboplatin, doxorubicin and taxane alone or in combination used as the most common treatment regimes, alongside hormone therapies such as megestrol for Type I estrogen-responsive cancers [11].

Differences exist in the molecular pathogenesis of type I and II endometrial cancer [12,13] and links have been established to alterations in epigenetic processes such as DNA (de)methylation [14], histone (de)methylation or (de)acetylation [15,16]. Nieminen et al. [12], identified 24 tumor suppressor genes that were progressively hypermethylated during the development of type I endometrial cancer [14,17,18]. Similarly, treatment of endometrial cancer models using small chemical compounds that modify epigenetic processes can partially, or even fully, restore the normal expression levels of genes including the re-expression of inactivated tumor suppressor genes and deactivation of activated oncogenes such as RASS-F1A, MGMT, HAND2, PTEN and MLH1 [19,20]. This epigenetic plasticity and the deregulation of epigenetic processes in endometrial cancer have led to the evaluation of suberoylanilide hydroxamic acid (SAHA), currently approved for the treatment of lymphoma and myelodysplastic syndromes, in endometrial cancer clinical trials [21]. SAHA inhibits class I and II HDACs by binding to the Zn^2+^ chelation center in the enzyme and affects processes that lead to cell cycle arrest and apoptosis [22,23,24]. However, in Phase II studies, SAHA has demonstrated little to no efficacy either alone or in combination with standard anti-cancer treatments [25]. Several factors may cause the lack of HDAC response including low stability in the blood stream (two-hour elimination half-life) and an inability to accumulate adequate concentrations at the tumor site, a combination of increased drug efflux pump activity and low tumor penetration [26]. Furthermore, the pharmacology of SAHA is particularly complex as it can act as an enzyme inducer following repeated dosing, increasing HDAC levels and may modify its own kinetics [25].

Strategies to overcome such limitations include the development of nanovectors to increase drug stability and accumulation of drug-containing nanoparticles at tumor sites [27,28,29,30,31,32]. The improvement of safety and tolerability by nanoparticle-formulated drugs is exemplified by liposomal doxorubicin [33], where increased concentrations of the therapeutic agent accumulate specifically in the tumor [34], by virtue of the purported enhanced permeability and retention effect [35,36]. Previously, passive encapsulation and conjugations (prodrug approaches) of SAHA have been shown to improve the drug efficacy in melanoma and breast cancers, respectively [37,38].

Hyaluronic Acid (HA) is a naturally occurring polymer that consists of N-acetyl-d-glucosamine and d-glucuronic acid repeat units and is a ligand for the CD44 receptor [39]. HA has previously been used successfully as a targeting agent in nanoformulations. Notably, Tran et al., designed a SAHA solid lipid nanoparticle (SLN) with HA surface expression for improved cellular uptake in lung adenocarcinoma (A549) and tongue squamous cell carcinoma (SCC-7) [40]. Even though there are benefits to lipidic carriers for drug delivery, SLNs have a several disadvantages including non-compatibility with hydrophilic agents, gelation of SLN suspensions, low loading capacities, high costs (comparative to a polymer alternative) and the increase in SLN particle size over time [41,42].

Polymers provide an excellent platform for nanovector systems, with many polymer nanoparticle developments reported for the treatment of solid tumors [43,44]. Pluronic^®^ F-127 is a water soluble triblock copolymer consisting of poly(ethylene oxide)-poly(propylene oxide)-poly(ethylene oxide) repeats (PEO101-PPO56-PEO101) that possess a low toxicity in vivo, and is FDA approved [45]. F127 nanoparticles have been developed for the controlled release of therapeutic drugs, peptides and proteins [46,47,48] and with carboxylic groups to mediate the addition of tumor targeting moieties such a folate [49,50]. HA is a ligand for the CD44 receptor [39,51] that is found at low levels on the surface of epithelial cells but is highly expressed in endometrial tumors, playing a key role in tumor development and progression [52,53]. In vivo studies have demonstrated that HA functionalized nanoparticles target CD44 over-expressing tumor sites [54,55], and exhibit enhanced stability in the bloodstream [40]. Previously, Pluronic^®^ F-127-HA conjugates have been used to form macromolecular hydrogels for slow drug release implants, however, there has been no application of HA-pluronic as discrete nano-micelles for drug delivery [56,57].

Here we demonstrate the efficient cytotoxicity of HA targeted SAHA nanoparticles (SAHA-NP-HA) in 3D endometrial cancer models. Pluronic^®^ F-127 polymer encapsulation enhanced SAHA cytotoxicity in vitro through delayed release kinetics in models of type I (Ishikawa) and type II (Hec50) endometrial cancer. Surface conjugation of HA further increased the cytotoxicity of SAHA in both cell types, that demonstrate distinct patterns of CD44 expression. The use of a 3D model revealed the marked improvement of SAHA-NP-HA versus the non-targeted counterpart, SAHA-NP. This finding reinforces the need to use 3D in vitro models during pre-clinical development of nanoformulations. Encapsulation did not affect the mechanism of action, as cellular phenotype, cell cycle checkpoint protein expression and histone H3 acetylation were similarly affected using the free and encapsulated drug. However, using this nano-encapsulation approach coupled with HA overcome the toxicity associated with SAHA in the treatment of endometrial cancer models.

## 2. Materials and Methods

Hyaluronic acid (MW 130 kDa) was purchased from LifeCore Biomedical (Chaska, MN, USA). 1-ethyl-3-(3-dimethylaminopropyl)-carbodiimide hydrochloride (EDC), 1-hydroxybenzotriazole hydrate (HOBt), DL-Dithiothreitol (DTT), Cysteamine hydrochloride, 2,2′-Dithiodipyridine, 4-Nitrophenyl chloroformate were purchased from Sigma-Aldrich (St. Louis, MO, USA). Dialysis membranes used for purification were purchased from Spectra Por-6 (MWCO 3500, Pomona, CA, USA). All solvents were of analytical quality. Nuclear Magnetic Resonance (NMR) spectroscopy experiments were performed with a Varian Mercury 300 MHz NMR Spectrometer (Palo Alto, CA, USA). All spectrophotometric analysis was carried out on Shimadzu UV-3600 plus UV-VIS-NIR spectrophotometer (Kyoto, Japan).

### 2.1. 2D Cell Culture

Both Ishikawa (ECACC 99040201 Public Health England, Salisbury, UK) a type I endometrial cancer (TIEC) model and Hec-50B (Cat-No 1145, JCRB Cell Bank, Japan) a type II endometrial cancer (TIIEC) model [58], were maintained in DMEM:F-12 (1:1) + GlutaMax™ full media (Cat-No 31331-028, Gibco, ThermoFisher Scientific, Loughborough, UK) supplemented with 10% fetal bovine serum (FBS), sodium bicarbonate 1 mM, sodium pyruvate 1 mM and 1% antibiotic-antimycotic solution in plastic culture flasks at 37 °C and 5% CO_2_ incubator (Nuaire, Playmouth, MN, USA). Cells were supplemented with full serum media every 2 days and passaged when confluent.

### 2.2. 3D Cell Culture

Spheroids were prepared for viability experiments using an ultra-low attachment 96 well plate [59]. 4 × 10^3^ cells per well were added to a 96-well Ultra-Low Attachment surface (ULA) microplate (Corning™ 4520, Corning, NY, USA) supplemented with stripped serum media. After 48 h treatment incubation, CellTiter-Glo^®^ 3D Cell Viability Assay reagents (Promega, Madison, WI, USA) were added following manufacturer’s instructions and luminescence recorded using a microplate reader (FLUOstar Omega, Aylesbury, UK) at RT. Spheroids for protein expression were prepared using the liquid overlay technique in clear 96 well plates [60]. Briefly, 96 well plates were conditioned for spheroid seeding by coating the bottom of each well with 2% agarose. The agarose was left to set for 1 h in the flow hood under ultraviolet (UV) light to ensure sterility. 6 × 10^4^ cells/mL were used to form the spheroids for western blot experiments. Spheroid morphology following treatments was assessed using Image J (FIJI). Images were taken using a Zeiss microscope at 40× objective with scale bar exported from the proprietary Zen software (Carl Zeiss Ltd., Cambridge, UK). Scale bar consistent exported images were analyzed in Image J (FijiLOCI, University of Wisconsin, Madison, WI, USA), setting measurements to include area, perimeter and shape descriptors using the particle analyzer function.

### 2.3. Confocal Laser Microscopy Analysis

Cells (2 × 10^4^ cells per well) were grown in 8 wells imaging chamber with microscope slides (Labtek, Brendale, Australia) and grown to 80% confluency. Cells were washed with PBS and fixed with 4% paraformaldehyde (PFA) for 20 min at 4 °C. Fixing solution was extensively washed out with PBS and 0.1% of Triton-X100 was added to the well and incubated for 5 min at 4 °C. After a wash step with PBS, a block buffer (3% BSA in PBS) was added and the plate incubated for 30 min. Wells were washed with PBS and then the primary antibody, which is diluted in block buffer was added to the plate and incubated 1 h or overnight at 4 °C. The primary antibody used was a rat anti-human monoclonal antibody CD44/H-Cam (Cat-No MA4400, Thermo Fisher Scientific, UK) (1:300 dilution). Primary antibody was washed out with 4 washes with PBS, then the secondary antibody was added and incubated for 1 h at room temperature. For the secondary antibody goat anti-rat IgG-FITC (Cat-N0 sc-2011, Santa Cruz, UK) was used. Following 4 washes with PBS, DAPI (Cat-No R37606, Thermo Fisher Scientific, UK) was added to the well and the plate analyzed using a Zeiss LSM 710 confocal microscope. Zen lite 2012 imaging software (Carl Zeiss Ltd., UK) was used for imaging analysis. For each antibody used in the experiments, specific parameters were set in the confocal microscopy, such as time of exposure and gain, to ensure that each slide were analyzed in the same way. Confocal quantification was performed using ImageJ software.

### 2.4. Synthesis of Thiolated HA (HA-SH)

HA-SH conjugates were synthesized by coupling dithiobis (propanoic hydrazide) (DTPH) using carbodiimide chemistry to the carboxylic groups of HA and subsequently reducing the disulphide bond using dithiothreitol (DTT). Briefly, 1 equivalent HA (400 mg, 1 mmol with respect to the disaccharide repeat units) was dissolved in 120 mL of deionized water followed by the addition of 1 equivalent 3,3′-dithiobis (propanoic hydrazide) (DTPH 238.3 mg, 1 mmol) and 1 equivalent HOBt (153 mg, 1 mmol) and stirred at room temperature until the reaction becomes homogeneous. Thereafter, the pH of the reaction mixture was adjusted to 4.7 by careful addition of 1 M NaOH and 1 M HCl. Finally, 0.25 mmol EDC·HCl (48 mg, 0.25 equivalent) was added and allowed to stir overnight. Then the reaction mixture was loaded into a dialysis bag (Spectra Por-6, MWCO 3500 g/mol) and dialyzed against dilute HCl (pH = 3.5) containing 100 mM NaCl (4 × 2 L, 48 h) and then dialyzed against deionized water (2 × 2 L, 24 h). The solution was lyophilized to obtained as white fluffy material. The degree of hydrazide modification was estimated to be 10.5% (with respect to the disaccharide repeat units) as determined by trinitrobenzene sulfonic acid (TNBS) assay by measuring absorbance at 500 nm, using UV spectroscopy [61].

In the second step, the lyophilized HA-DTPH derivative was dissolved in 100 mL deionized water and pH of the solution was adjusted to 9 with 1 M NaOH. Subsequently, dl-Dithiothreitol (DTT, 124 mg, 0.8 mmol) was added dropwise to the solution. The mixture was stirred overnight, after which the solution was transferred to a dialysis bag (Spectra Por-6, MWCO 3500 g/mol) and dialyzed against deionized water (3 × 2 L, 24 h). The dialyzed solution was lyophilized to give white fluffy thiol-modified HA (HA-SH). The concentration of thiol groups was estimated to be 7% (with respect to the disaccharide unit) by Ellman’s assay using the extinction coefficient of 14,150 M-1cm-1 at 412 nm using UV spectroscopy, FT-IR spectra of material given in supplementary material (Figure S1c) [62].

### 2.5. Synthesis of Pluronic^®^ F-127 Micelles

Pluronic^®^ F-127 (NP) and thiol-terminated Pluronic^®^ F-127 (NP-S-S) micelles were fabricated using thin-film hydration method described by Caldwell et al. [63]. The polymer constituent and 10 wt/wt.% SAHA (Cayman Chemical Company, 10009929, Ann Arbor, MI, USA)/propidium iodide (Sigma-Aldrich, P4170-10MG) were dissolved in acetonitrile (ACN). The acetonitrile was removed by rotary evaporation at 65 °C and under 226 mbar for 1 h, and the flask placed into a desiccator under vacuum overnight, to ensure the film was void of all moisture and residual copolymer matrix acetonitrile removed. Prior to hydration, samples were heated to 65 °C in a water bath under mild rotation for 1 h until the sample resembled a viscous thin film coating the flask. NPs were hydrated in deionized water and returned to heated rotation for 30 min to ensure maximum micelle formation and drug loading. HA-thiol was added in excess (2:1) to NP-S-S and mixed using a magnetic stirrer at 700 rpm for 1 h to form NP-HA; prior to use HA-thiol was sterilized under UV for 1 h. An 0.22 or 0.45 μm filter (Millipore, Burlington, MA, USA) was applied to SAHA-NP and SAHA-NP-HA to remove non-encapsulated drugs agglomeration. 

Synthesis of thiolated Pluronic^®^ F-127 (NP-S-S-Py).

### 2.6. Activation of Pluronic F127

The synthesis of pluronic F127 functionalized with terminal disulphide pyridyl was performed following the reported procedure [64]. Briefly, the terminal PEG diols of pluronic F127 was activated using 4-nitrochloroformate. The degree of activation was found to be 78%, determined spectrophotometrically by measuring the amounts of 4-nitrophenolate ions released in the alkaline solution by measuring the absorbance at 402 nm using a molar extinction coefficient of 18400 cm-1M-1.

### 2.7. Synthesis of Pyridyl Disulphide Ligand (2-(2-Pyridyldithio) Ethylamine)

Cysteamine hydrochloride (2.288 gm, 20.1408 mmol) was dissolved in methanol (17.5 mL) followed by the addition of glacial acetic acid (1.6 mL). This solution was then dropwise added to a stirred solution of 2,2-dithiopyridine (8.815 gm, 40.2817 mmol) in methanol (41.6 mL). Reaction mixture was stirred for 48 h at room temperature and product was precipitated from stirred diethyl ether (200 mL). The product was dissolved in a small volume of methanol and was again precipitated by diethyl ether to afford a white solid compound. The 1H NMR of the pyridyl disulphide product was consistent with the reported data, see supplementary material (Figure S1a) [64].

### 2.8. Synthesis of F127 Pyridyl Disulphide Derivative (NP-S-S-Py)

Activated pluronic F127 (1 gm, 0.069 mmol) dissolved in 10 mL DCM was reacted with extracted disulphide ligand (154 mg, 0.69 mmol) dissolved in 1 mL DCM. The reaction mixture was refluxed overnight. It was then concentrated and diluted with methanol: water (1:1, *v*/*v*, 10 mL) and dialyzed (membrane MWCO 3500 Da) against 2 L deionized water for two days. The product was obtained as white fluffy material after lyophilization. The modification was determined using UV (343 nm, molar extinction coefficient of 8060 cm-1M-1). The UV absorbance of product (1 mg/mL in PBS buffer at pH 9) was measured before as well as 10 min after the addition of 0.1 mL of DTT (15 mg/1000 mL in PBS at pH 9). The percentage modification was found to be 78%, FT-IR spectra of material given in supplementary material (Figure S1b).

### 2.9. Size and Zeta Potential Analysis with Dynamic Light Scattering (DLS)

Polymeric micelle size and zeta potential were determined using Zetasizer Nano ZSTM (Malvern, UK), using a particle concentration of 100 μg/mL. A series of data indicating size, zeta-average, polydispersity index was generated. All data is presented as average and standard deviation, from a minimum of three independent biological repeats.

### 2.10. Atomic Force Microscope (AFM)

10 μL nanoparticle aliquots were spotted on mica substrates at a concentration of 100 μg/mL (AGG250-1, Agar Scientific, Stansted, UK) and dried at RT. Sample topography was obtained in air using a Bruker BioScope Catalyst (Bruker Instruments, Santa Barbara, CA, USA) AFM. Bruker ScanAsyst-Air cantilevers were used, with a nominal spring constant of 0.4 N/m and a nominal resonant frequency of 70 kHz. All imaging was conducted using Peak Force Tapping (PFT) in ScanAsyst Mode. Images were processed with first-order flattening and planefit using Bruker Nanoscope Analysis 1.5, the height of the particles was calculated using freeware AFM software WsXM 5.0 [65].

### 2.11. HPLC-UV Quantification

An Agilent detector equipped with autosampler and a C18 reversed-phase column (Xbridge C18, 130 Å, 3.5 μm, 2.1 mm × 150 mm) was ran isocratically at room temperature (RT). All samples were diluted using acetonitrile and loaded into the 0.3 mL polypropylene, 9 mm thread, screw thread vials (Sigma, 29377-U); mobile phase throughout analysis was 0.1% formic acid:acetonitrile 78:22 [66]. A flow rate of 0.25 μL/min and sample injection volume of 2 μL gave a 7 min analyte retention time. Following elution from the column, the sample was run through the UV/vis detector recording at 241 nm. Nanoparticle samples were diluted 1:10, 1:100 and 1:200 to ensure measurements were occurring within the range of the standard curve. Encapsulation efficiency was determined using the equation below (1), loading capacity was calculated using Equation (2).

Encapsulation Efficiency (EE%) = mass of drug in particles/mass of drug used in fabrication(1)

Loading Capacity (LC%) = mass of encapsulated drug/mass of nanoparticle material(2)

### 2.12. Drug Release Profile

Cellulose dialysis sacks with a molecular weight cut-off pore size of 12 kDa (Sigma Aldrich, D6191-25EA, St. Louis, MO, USA) were used to monitor drug release profiles over 80 h. Dialysis sacks were soaked in PBS buffer overnight, 1.5 mL of sample was loaded into the cellulose tubing and placed in a beaker containing 20 mL of PBS and a small magnetic fly. The buffer was mixed continually throughout the experiment by stirring at 300 rpm. For the first 2 h of dialysis, 1 mL samples were taken at 30 min intervals, for the remainder of the first 48 h samples were taken every 60 min. Following each sampling, 1 mL of fresh PBS was reintroduced to maintain a total volume of 20 mL. Samples were diluted in ACN to dissociate polymer micelles and frozen until analysis.

### 2.13. Cellular Penetration

2D NP cellular internalization was assessed using fluorescent dye (propidium iodide) loaded NP treatments, visualized with Zeiss LSM 710 confocal microscope to determine cellular localization and In Cell 6000 Analyzer (GE healthcare, Amersham, UK) for high throughput analysis of NP containing PI frequency. See supplementary materials for additional information.

3D NP internalization was assessed using flow cytometry conducted using an Amnis^®^ CellStream^®^ benchtop flow cytometry system equipped with 375 nm, 405 nm and 488 nm lasers, a dedicated side scatter laser (785 nm) and dedicated forward scatter LED (450 nm) coupled to a filter stack for spatial separations of photons (456/51, 528/46, 583/24, 611/31, 702/87 and 773/56 nm). 3D cell cultures were treated with NP treatments loaded with fluorescent dye propidium iodide for 48 h, 24 spheroids were used per treatment. Spheroids were collected and disaggregated via accutase (Sigma Aldrich; A6964-500mL) incubation for 10 min at 37 °C with mechanical pipetting every minute. Cells were pelleted and resuspended in flow cytometry buffer (1× dPBS supplemented with 3% FBS, 2mM EDTA and 2mM NaN_3_) and nuclei were co-stained using Hoescht stain (Thermo Fisher Scientific, H3570). Samples were run at 14.64 μL and fluorescence was measured on channels A2 (405/528 nm) for Hoescht and C6 (488/702 nm) for PI.

### 2.14. Chromatin Immunoprecipitation (ChIP)

Ishikawa and Hec50 cells were grown in stripped media for 24 h in T25 flask. SAHA treatments at 2.5 μM were added for 48 h. Following that time, approximatively 5 × 10^6^ cells for each cell line were processed for ChIP analyses following manufacturer’s instructions (Chromatrap^®^ ChIP-seq, Norfolk, UK) [67]. The following antibodies were used; Active Motif, AcH3 (#39140, ChIP-seq); Sigma-Aldrich, IgG rabbit (#12-370, ChIP-seq), IgG mouse (#12-371, ChIP). PCR was used as the endpoint assay to analyze the ChIP DNA, using a BioRad CFX real time PCR machine (BioRad, Watford, UK). The primers sequences used were P21 Forward 5′-CCCACAGCAGAGGAGAAAGAA-3′, P21 Reverse 5′-CTGGAAATCTCTGCCAGACA-3′; P53 Forward 5′-AGACCAGCCTGACCAATA-3′, P53 Reverse 5′-GCTCCCGGAAACATCTCAC-3′. The human GAPDH primers present in the Chromatrap^®^ ChIP-seq kit was used as a positive control. The ratios of the signals in immunoprecipitated DNA versus input DNA was calculated as previously shown [68].

### 2.15. 2D/3D Cell Viability Assays

Cell viability assays were performed in 2D using Realtime-Glo™ MT Cell Viability RT-Glo (Promega, UK, G9711) and in 3D using Celltiter-Glo 3D (Promega, UK, G9682). Both methods were performed according to the manufacturer’s instructions [69,70]. Readings were taken using a FLUOstar Omega microplate reader at 0-, 24-, 48- and 72 h time points. For 3D experiments, only 48 h and 72 h timepoint was investigated. The spectrophotometer was heated to 37 °C prior to readings. All SAHA-NP preparations were compared to free drug SAHA and all data was normalized against the control cell line without any treatment. All treatments were 2.5 μM unless otherwise stated.

### 2.16. Protein Blotting and Antibodies

Total protein lysates (20 μg) were resolved on a precast 4–20% polyacrylamide gels (mini-PROTEAN^®^ TGX stain-free™ gels, 456-8094), transferred and immobilized onto polyvinylidene fluoride (PVDF) membranes (mini format Trans-Blot^®^ Turbo™ transfer pack, 170-4156), incubated for 60 min at room temperature in blocking solution (TRIS-buffered saline containing 5% BSA and 0.1% Tween 20), followed by an overnight incubation in primary antibodies at 4 °C at 1:1000 dilution. The following antibodies were used: Cell Signaling (Danvers, MA, USA): p21 (#2947s; 1:1000), p53 (#9282s; 1:1000), Santa Cruz: GAPDH (# sc-47724; 1:1000). Abcam (Cambridge, MA, USA): E-Cadherin (#1416; 1:1000), N-Cadherin (#18203; 1:1000), GE Healthcare Lifescience: anti-rabbit (#10794347, 1:2000), anti-mouse (#10094724, 1:2000). Membranes were then washed three times and incubated with horseradish peroxidase–conjugated secondary antibodies for 1 h at 1:2000 dilution.

### 2.17. Immunofluorescence and Antibodies—2D

Cells (2 × 10^4^ cells per well) were grown in 8 wells imaging chamber with microscope slides (Ibidi, Gräfelfing, Germany) and analyzed using a Zeiss LSM 710 confocal microscope. Wells were imaged at 80% confluency or after 48 h treatments. At treatment duration or 80% confluency, cells were washed with warm PBS and fixed with 10% formalin (Methanol-free, Merck, Kenilworth, NJ, USA) for 5 min at room temperature, washed 3 times with PBS and permeabilized using 0.1% triton (X100–100ML; Sigma Aldrich) for 5 min at 4 °C, followed by 3 washes. Samples were blocked using 3% BSA in PBS for 1 h at room temperature. Followed by an overnight incubation in primary antibodies at 4 °C at 1:100 dilution. Samples were washed 3 times and incubated with 1:200 dilution of secondary antibodies for 1 h at room temperature. The follow antibodies were used: Santa Cruz: CD44 (HCAM F-4; sc-9960), N-cadherin (sc-5997); Abcam: E-cadherin (ab40772); Thermo Fisher: Anti-mouse Alexa 488 conjugate (A11001); Anti-rabbit Alexa 594 conjugate (A32740). Nuclei were co-stained with Hoescht stain, incubated in a 1:2000 dilution in PBS for 5 min at room temperature. Samples were washed thoroughly prior to imaging.

### 2.18. Immunofluorescence—3D

50 spheroids (4 × 10^3^ cell per spheroid) per condition were seeded on agarose plates, left for 48 h to form then treated for 72 h prior to collection and processing in 15 mL falcon tubes. Unless otherwise stated, samples were agitated using a rocker set to 30 rpm during incubations. Spheroids were fixed and permeabilized for 3 h at 4 °C and using 4% PFA, 1% Triton in PBS. Spheroids were dehydrated at 4 °C using an ice-cold methanol series (25%, 50%, 75% and 95%; Sigma Aldrich, 34860-2.5L-R), incubations were 20 min per stage. Following this, a pure methanol incubation was conducted for 1 h. Rehydration of the spheroids was conducted by reversing the previous methanol series, also for 20 min per stage. Samples were thoroughly washed (PBS with 0.1% triton, PBST) prior to blocking in 3% BSA, 0.1% triton overnight at 4 °C. Duplicate PBST washes were carried out prior to primary antibody incubations (same antibodies as above), incubations were 72 h at 4 °C. Primary antibody was removed by washing in PBST for 24 h prior to the 24 h secondary antibody incubation. Finally, samples were washed thoroughly with PBST, nuclei were co-stained using Hoescht stain (1:2000) for 25 min at room temperature. Spheroids were moved to 8-well chamber slides for imaging.

### 2.19. Statistical Analysis

Data were normalized against the respective cell line without any treatment. Statistical analyses used to in this study to validate results, unless stated otherwise, were pairwise student’s *t*-Tests and ANOVA where treatments were compared against the free drug only. Significance is indicated on graphs or tables, when appropriate in the following fashion: *p* ≤ 0.05 denoted by *, *p* ≤ 0.01 by **, *p* ≤ 0.001 by *** and *p* ≤ 0.0001 by ****.

## 3. Results and Discussion

### 3.1. Effective SAHA Dose Determination for Type I and II Endometrial Cancer Models

**Figure 1:**
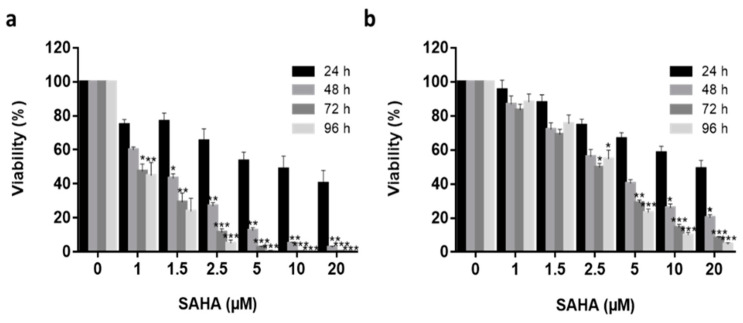
Free SAHA drug cytotoxicity in in vitro Endometrial cancer models. Increasing concentrations (1 μM to 25 μM) of free SAHA were added to both Ishikawa (**a**) and Hec50 (**b**) cells over a 96 h period (24 h—black columns, 48 h—medium grey, 72 h—dark grey, 96 h—light grey) and viability quantified using Real Time Glo viability test (*n* = 3), independent biological experiments. All data was normalized to the untreated control cells. Statistical significance is indicated on graphs: *p* ≤ 0.05 denoted by *, *p* ≤ 0.01 by ** and *p* ≤ 0.001 by ***.

Ishikawa (type I) and Hec50 (type II) endometrial cancer cell lines [58] were used to determine an effective treatment concentration for SAHA prior to its encapsulation in Pluronic^®^ F-127 vectors. Ishikawa and Hec50 cells were treated with an increasing dose of SAHA concentration (1 μM to 20 μM) over 96 h (Figure 1) and found to have different response profiles to SAHA treatment, with Ishikawa cells being much more sensitive to the drug [21,71,72]. No significant reduction in Ishikawa cell viability was observed following treatment with 1 µM SAHA after 48 h (*p* > 0.05), the half-maximal inhibitory concentration (IC50) was observed at 1.5 µM (*p* > 0.05) and IC99 at 20 µM (*p* < 0.001) treatment. Hec50 cells demonstrated approximate IC15, IC45 and IC95 following 1 µM, 2.5 µM (*p* < 0.05) and 20 µM (*p* > 0.001) treatments, respectively. Using the 72 h data series, the IC50 was determined to be 1.2 µM and 2.7 µM for Ishikawa and Hec50, respectively. Having established the response profiles to SAHA exposure, 2.5 μM was selected for all further treatments, as this was the lowest concentration where a significant reduction in viability was observed for both cell types.

**Figure 2:**
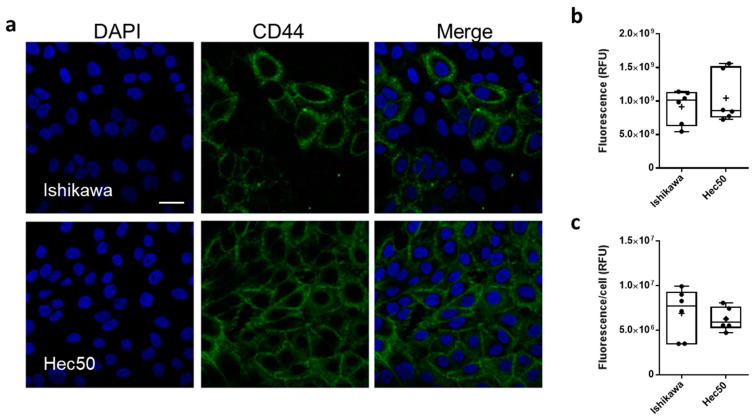
CD44 expression in endometrial cancer models. Representative immunofluorescence images showing CD44 expression (FITC, green) in Ishikawa and Hec50 cells (**a**) nuclei where stained with DAPI (blue), scale bar is 30 μm. Relative fluorescence units (RFU) values obtained from ImageJ analysis of immunofluorescence images (**b**), RFU per cell based on nuclei count (**c**) where each point plotted is value from each field analyzed.

CD44 protein expression was characterized in both Ishikawa and Hec50 cell lines, using confocal laser scanning microscopy (CSLM; Figure 2). CD44 was shown to be expressed throughout the Ishikawa population with a mean relative fluorescence (RFU) of 9.15 ± 1.02 × 10^8^ and a mean cellular RFU of 6.88 ± 1.4 × 10^6^ (Figure 2b,c and Figure S2). In comparison, only a portion of Hec50 cells assessed were shown to be CD44+, with a whole population RFU of 1.04 ± 0.15 × 10^9^ and a cellular RFU of 6.26 ± 0.52 × 10^6^. Interestingly, this heterogenous expression of CD44 still amounts to a similar mean expression to that shown in the homogenous Ishikawa population, due in part to the number of high expressing cells across the monolayer.

### 3.2. Physicochemical Characterisation of Functionalized and SAHA-Loaded Pluronic^®^ F-127 Nanoparticles

Pluronic^®^ F-127 nanoparticles (NP) and thiolated pluronic nanoparticles (NP-S-S) were fabricated using thin-film hydration [50]. HA was conjugated to particles post-fabrication by HA-thiol treatment, to obtain HA-coated NPs (NP-HA) [73]. Briefly, a disulphide bridge was introduced by activating the PEG-diols with 4-nitrochloroformate followed by reacting with 2-(2-pyridyldithio) ethylamine to obtain NP-S-S. The efficiency of this pyridyl functionalization was 78% following reduction. Separately, HA-thiols were synthesized by conjugating 3,3′-dithiobis (propanoic hydrazide) followed by reduction with DTT. The degree of thiol modification was estimated by Ellman’s assay and was found to be 7% with respect to disaccharide repeat units. SAHA payloads were incorporated into the micelles by co-dissolution prior to thin-film formation. DLS measurements of empty NP gave sizes of 29.1 ± 2.1 nm, particle size increased to 35.7 ± 3.6 nm after drug incorporation (SAHA-NP) and the addition of HA to the surface of the particle (SAHA-NP-HA) gave a further increase to of 251.6 ± 22.0 nm (Table 1).

**Figure 3:**
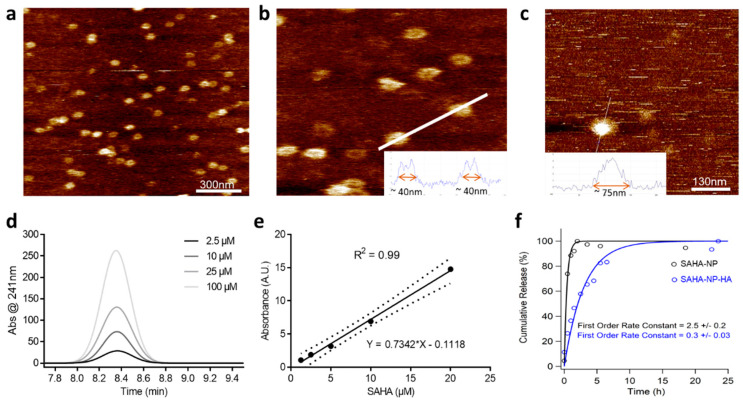
Nanoparticle AFM imaging. F127 nanoparticles were deposited on a mica substrate and analyzed by AFM. (**a**) NP topography showing the round shape. (**b**) Vertical line profile calculated along the solid white dotted line showing particle height around 40 nm. Scale bar 300 nm. (**c**) NP-HA image showing size and topography. Encapsulation efficiency and release profile HPLC chromatogram and calibration curve. SAHA encapsulation efficiency was calculated relative to a standard curve constructed from increasing SAHA concentrations from over range of 2.5 to 100 μM (absorbance at 241 nm and retention time = 8.34 min) in DMSO solution, measured by HPLC (**d**). Calibration curve (**e**) and linear fitting are representative of that constructed independently for each particle preparation. The purity (based on AUC) was >95%. Cumulative passive SAHA release profiles from nanoparticles (**f**). Cumulative percentage as a proportion of the released drug, not total drug encapsulated. Data generated using LC-UV analysis. SAHA-NP (black line) and SAHA-NP-HA (blue line) release profiles with corresponding first order rate constant.

High resolution analysis was undertaken by using AFM peak-force tapping mode imaging, giving more accurate size determination than is possible with DLS [74,75,76,77,78]. The spherical geometry of intact particles was clearly demonstrated, with diameters of 40 ± 3 nm and 75 ± 7 nm for SAHA-NP and SAHA-NP-HA, respectively (Figure 3a,b). These size ranges and particle morphology were confirmed using TEM (See Figure S3). Image analysis of over 250 particles per formulation gave mean sizes of 20.3 ± 4.6 nm for SAHA-NP and 43.8 ± 18.7 nm for SAHA-NP-HA. These sizes are notably smaller than values from AFM data and the standard deviation is much larger. Previous comparisons of six different characterization techniques found that TEM under-estimated NP size in comparison to both DLS and AFM, potentially an artefact of the staining and drying process (which could lead to micelle branches collapsing) required for TEM analyses [79]. F127 micelles displayed uniformity after drug encapsulation as shown in the PDI measurements, where SAHA-NP particles exhibited a PDI of 0.354 and the empty NP 0.243. HA functionalization resulted in a significant decrease in zeta potential to −14.23 ± 3.29 mV compared to SAHA-NP (−0.197 ± 0.15 mV) (Table 1). SAHA encapsulation efficiency was 42% for SAHA-NP-HA (Table 1) and was 2.5-fold greater than SAHA-NP (18%; *p* < 0.05). SAHA-NP-HA had an enhanced loading capacity (LC) of 9.7% (wt./wt.%), a 16-fold increase compared to SAHA-NP (0.6% wt./wt.%, *p* < 0.01) and demonstrated a significant increase in LC compared to comparable reported drug delivery systems (DDS) where SAHA encapsulation into HA-coated particles was only 1.9% [40].

Passive drug release at physiological salt concentrations (151.5 mM PBS; Figure 3f) determined a consistent release was established at 7.5 h for SAHA-NP and 25.0 h for SAHA-NP-HA, a delayed release between SAHA-NP and SAHA-NP-HA of 17.5 h. Maximal drug release was at 2 h for SAHA-NP and 24.5 h for SAHA-NP-HA. Presented (Figure 3e,f) are cumulative percentage as a proportion of the released drug only, not total drug encapsulated. All formulas were shown to retain at least 94% of payload over the time period investigated. The slow and sustained release of SAHA for all formulations, evidenced by plateau in Figure 3f, demonstrated the advantage of nano-formulation in terms of the efficient stabilization of hydrophobic SAHA at the PPO core and supported the absence of any burst release. Drug release modelling of SAHA-NP and SAHA-NP-HA gave first-order release constants of 2.5 +/− 0.2 and 0.3/−0.03, indicating the addition of HA to the particle surface altered release kinetics. It is likely that the slower release rates observed were due in part to the super-saturation of hydrophobic SAHA at the higher concentrations encapsulated in these particles [80]. In addition, HA is a relatively large polymer and creates a rate-limiting barrier [81,82] likely resulting in increased SAHA stability in free circulation and prolonged release. Over the 72 h period the mass of SAHA released from each formula was 28.1 µg (SAHA-NP) and 39.6 µg (SAHA-NP-HA) less than 5.5% of total drug cargo for all formulations, demonstrating a stable DDS that retains the majority of the encapsulated cargo. This data is a significant improvement on similar lipid-based systems, [38] where HA-SLN encapsulation of SAHA demonstrated concerns of rapid drug release where 50% of drug had been expelled in the first 10 h.

### 3.3. NP Cellular Uptake and Biocompatibility

**Figure 4:**
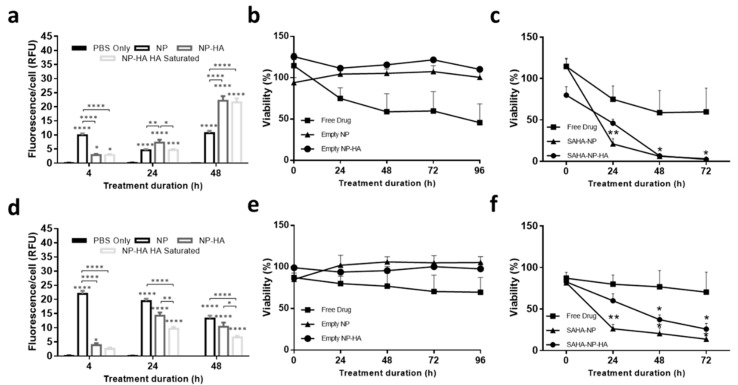
Nanoparticle uptake in 2D endometrial cancer models. Quantified uptake in Ishikawa (**a**) and Hec50 (**d**) per cell from 3 repeats, 9 fields per repeat, treatments were NP (black), NP-HA (dark grey) and NP-HA following HA saturation (light grey) compared to negative control PBS only (black with black fill). All treatments were 2.5 μM. Statistical analyses shown are results from one-way ANOVAs compared against PBS only unless indicated otherwise. Nanoparticle systems cytotoxicity in in vitro endometrial cancer models. RT-Glo viability curves for free drug (squares), Empty NP (triangle) and Empty NP-HA (circle) at 2.5 μM concentration for Ishikawa (**b**) and Hec50 (**e**) over a 96 h period (*n* = 4). All data was normalized to the untreated control cells. Encapsulated SAHA-NP drug systems cytotoxicity in vitro endometrial cancer models. Monolayer cellular viability using Real Time Glo viability test (*n* = 3) of SAHA Free Drug (square), SAHA-NP (triangle) an SAHA-NP-HA (circle) at the concentration of 2.5 μM for Ishikawa cell line (**c**) and Hec50 (**f**). All data was normalized to the untreated control cells. Statistical significance is indicated on graphs: *p* ≤ 0.05 denoted by *, *p* ≤ 0.01 by ** and *p* ≤ 0.0001 by ****.

To quantify nanoparticle uptake Ishikawa and Hec50 cells were exposed to 2.5 μM nanoparticles loaded with propidium iodide (PI). The fluorescence is only observed following internalization thus distinguishing between internalized and adsorbed nanoparticles [83,84]. Uptake was assessed at 4, 24 and 48 h, with significant uptake observed at all timepoints (Figure 4a,d; Figure S4a). In Ishikawa (Figure 4a), NP(PI) uptake did not increase from the original fluorescence seen at 4 h (mean RFU 9.9 ± 0.7 and 10.7 ± 0.8 at 4 and 48 h, respectively; ns), indicating that uptake and metabolism was occurring at similar rates. Cells treated with NP-HA show an initial lag in uptake (mean RFU 2.9 ± 0.4, 4 h) in comparison to the NP treatment, however, by 48 h a much greater degree of internalization, 22.1 ± 1.6, was observed, (*p* < 0.0001). Example of each channel plus merge given in Figure S4b and z-stack of Ishikawa showed positive signal for NPs across entirety of cell, with an increased affinity for nuclear localization (Figure S4c) In Hec50 cells, NP uptake over time decreased from 22.0 ± 1.1 at 4 h to 13.2 ± 1.0 at 48 h, whereas NP-HA uptake improved over time from 3.9 ± 0.7 at 4 h to 10.3 ± 1.5 at 48 h. Distinct temporal cellular uptake patterns across different cell lines has previously been reported with F127 micelles [78]. Whilst the internalization level is lower for Hec50 treated with NP-HA than NP, the particle is significantly larger (an increase from 30 to 251 nm). In addition, the inclusion of the HA moiety has been shown to improve circulation, stability and targeting in vivo, preventing non-specific uptake. Moreover, due to improved encapsulation efficiency seen in the functionalized particle, less particle needs to be internalized to achieve the same drug effect.

To investigate the ability of the HA moiety to mediate uptake, cells were treated with a saturating level of HA [39] prior to NP-HA treatment. HA saturation, resulted in significantly decreased uptake levels, in both cell lines (Figure 4a,d). In Ishikawa, uptake was decreased from 7.3 ± 1.0 to 4.7 ± 0.3 (*p* < 0.05) at 24 h, this effect was not retained at 48 h. Hec50 showed consistent behavior at both 24 and 48 h, with uptake decreasing from 14.2 ± 1.2 to 9.6 ± 0.7 (24 h; *p* < 0.01) and 10.3 ± 1.5 to 6.6 ± 0.5 (48 h; *p* < 0.05), respectively. This difference in behavior may be due, in part, to cellular levels of hyaluronidase. This enzyme facilitates the hydrolysis and metabolism of hyaluronic acid [85]; Ishikawa, such as many type I ECs, retain hyaluronidase functionality. In contrast Hec50 and type II ECs are known to downregulate or silence this enzyme [85,86,87].

### 3.4. Micelle Encapsulation Enhances SAHA Cytotoxicity

Ishikawa and Hec50 cells were grown in the presence of empty nanoparticles or NP-HA particles, with no significant increase in cellular toxicity observed (Figure 4b,e; *p* > 0.05), demonstrating the biocompatibility of the polymer with or without the HA moiety attached. The cytotoxic effect of SAHA-NP and SAHA-NP-HA was evaluated on Ishikawa cell monolayers (Figure 4c), and both SAHA-NP and SAHA-NP-HA were found to significantly reduce cell viability by 94% (*p* < 0.05) and 97% (*p* < 0.05), respectively, after 48 h. Both NP formulations were much more effective compared to free SAHA (41% reduction/IC40, Figure 4c). After 72 h cell viability had continued to decrease, a reduction of 97% and 98% was observed for SAHA-NP (*p* < 0.05) and SAHA-NP-HA (*p* < 0.05). Free drug SAHA after 72 h essentially had no further reduction in viability by 39%, possibly indicative of recovery and reflecting that free drug has poor long-term stability. In Hec50, following 48 h (Figure 4f) cellular viability was reduced by 79% (*p* < 0.05) following SAHA-NP exposure, compared to only 23% for the free drug (IC25) (Figure 4). Cell viability was further decreased with SAHA-NP after 72 h, significantly lowered by 86% (*p* < 0.05) compared to only 30% with the free drug (IC30). Encapsulation of SAHA substantially improved efficacy, however HA incorporation into the NP system did not enhance the efficacy of the SAHA-NP in 2D cell models, where cell viability decreased by 63% after 48 h and 74% after 72 h, respectively.

### 3.5. SAHA-NP Treatment Alters Histone H3 Acetylation at the Promoters of p21 and p53

**Figure 5:**
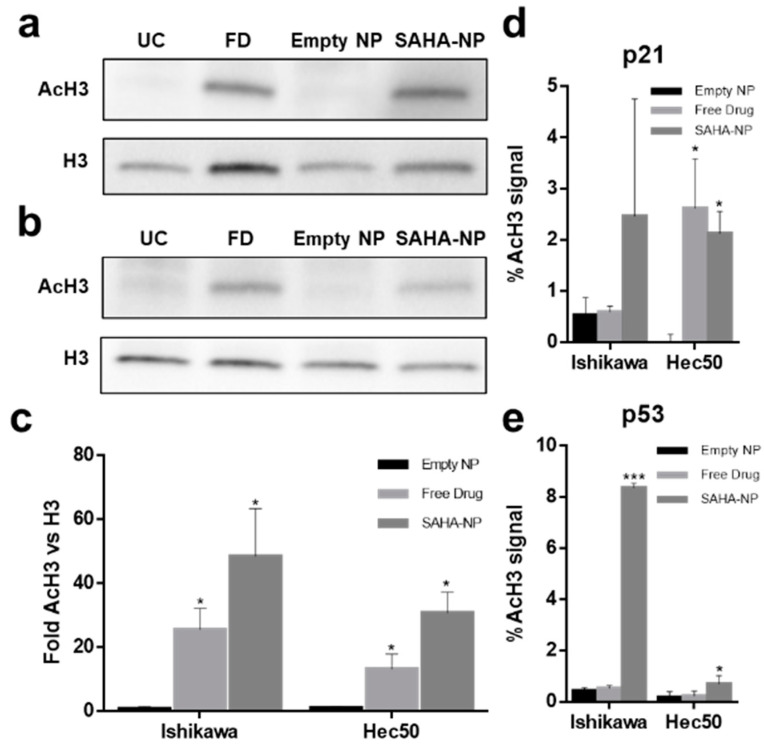
Mechanism of action of SAHA encapsulated and free SAHA drug in endometrial cancer cells. Protein blot analysis (AcH3 and H3) to demonstrating the SAHAs mechanism of action in Ishikawa (**a**) and Hec50 cells (**b**) (original images can be found at Figure S9), and protein density analysis of AcH3 (**c**) following 48 h treatments (*n* = 3). Treatments tested; empty nanoparticle (black), free SAHA (light grey) and SAHA-NP (dark grey), all treatments were 2.5 μM. All data are normalized to the untreated control cells. Effect of SAHA on AcH3 binding to the p21 promoter (**d**) and p53 promoter (**e**) in endometrial cancer cells and promoter methylation. ChIP experiments, SAHA effect on binding of AcH3 antibody to the p21 and to the p53 promoter. Hec50 and Ishikawa cells were treated with empty nanoparticle (black), free SAHA (light grey) and SAHA-NP (dark grey) (*n* = 3). Statistical significance is indicated on graphs: *p* ≤ 0.05 denoted by * and *p* ≤ 0.001 by ***.

SAHA inhibits histone deacetylation resulting in increased histone (H3 and H4) acetylation levels [22] and affects cell cycle progression through restoration of p21 expression in both Ishikawa and Hec50 cells [23] while reducing the expression of p53 in Ishikawa cells [88,89]. To determine whether these molecular responses were altered following encapsulation, global levels of acetylated histone H3 (AcH3) were measured (Figure 5a–e) and SAHA-NP treatment was shown to induce an expected 2–3-fold increase in the global level of AcH3, as well as induce locus specific increase in acetylation for both p21 and p53.

**Figure 6:**
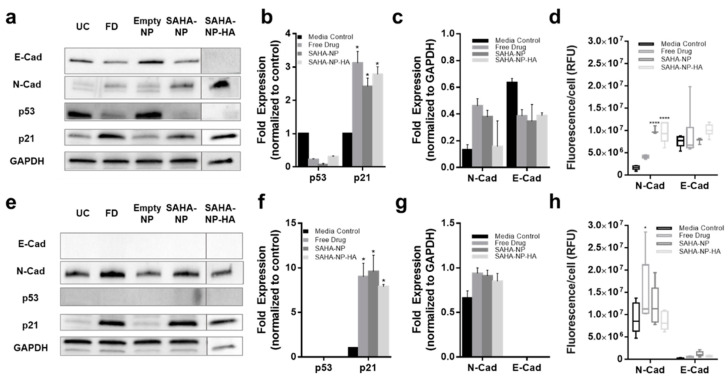
SAHA and SAHA-NP systems alter p21 and p53 expression and molecular in endometrial cancer cells. Protein blots for Ishikawa (**a**) (original images can be found at Figure S10) and Hec50 (**e**) (original images can be found at Figure S11), and densitometry for media control (black) and 2.5 μM SAHA (medium grey), SAHA-NP (dark grey) and SAHA-NP-HA (light grey) for 48 h for p53 and p21 in Ishikawa (**b**) and Hec50 (**f**) and E and N cadherin in Ishikawa (**c**) and Hec50 (**g**). * p53 and p21 are average and standard error, from a minimum of three independent repeats normalized to the untreated controls and GAPDH protein density. Cadherin values are average and standard error from a minimum of three independent repeats normalized to GAPDH protein density level only. Cadherin expression in endometrial cancer models. Immunofluorescence of E and N-cadherin in Ishikawa (**d**) and Hec50 (**h**) cell lines following 48 h treatments, relative fluorescence units (RFU) values obtained from ImageJ analysis of immunofluorescence images (*n* = 6), RFU per cell based on nuclei count. Statistical significance is indicated on graphs: *p* ≤ 0.05 denoted by * and *p* ≤ 0.0001 by ****.

These changes in histone acetylation levels contribute to altered protein expression. As expected, p53 protein expression was reduced after treatment with all SAHA-NPs in Ishikawa cells (Figure 6a,b), however as Hec50 are p53 null expression was undetected (Figure 6e,f). Conversely, in response to SAHA-NP treatments, p21 expression increased approximately four times more in Hec50 compared to Ishikawa, indicating a hyper-reactivation of p21 in this type II endometrial cancer model (Figure 6a,b,e,f). Epithelial–mesenchymal transition (EMT) status has been shown to be an important prognostic marker in endometrial cancer [90], and SAHA has been shown to induce a pro-migration phenotype [91,92,93]. N-Cadherin expression was upregulated following all SAHA treatments and E-cadherin was downregulated in Ishikawa following all treatments, an association seen in mesenchymal phenotypes [90]. Immunofluorescence analysis of N and E cadherin (Figure 6d,h and Figure S5a,b), corroborate protein blot analyses and show heterogeneity of N-cadherin upregulation in Ishikawa populations following SAHA treatment. Ratios of N:E (Figure S5c), show a change in Ishikawa from 0.2 to 0.5, 1.4 and 0.9 following free drug, SAHA-NP and SAHA-NP-HA, respectively.

### 3.6. HA Enhances Cytotoxicity of SAHA Nanoparticles in 3D Tumor Models

3D cellular models involve the establishment of complex cell-cell interactions as well as nutrient and oxygen gradients [94] allowing a more patho-physiologically relevant evaluation in drug development to be undertaken [95]. Whilst there is considerable variation between cell types, cells grown in spheroids are more akin to tumor cells in situ. The majority of cells in a spheroid, other than surface cells, leave the growth phase of cell cycle and become dormant due to contact inhibition [96]. This more closely reflects proliferation rates and metabolism of tumors in vivo. A comparative study on the growth properties of endometrial cancer cells in monolayers versus spheroids has shown that this method of cell culture leads to metabolism, proliferation and autocrine/paracrine signaling reflective of tumors in situ, traits that are not displayed in monolayer cultures [97].

**Figure 7:**
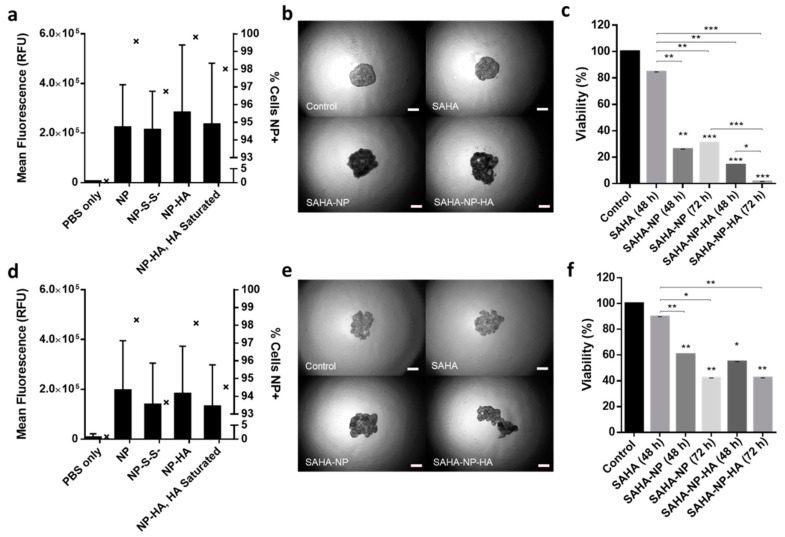
Nanoparticle uptake in 3D models. Quantified uptake in Ishikawa (**a**) and Hec50 (**d**) per cell, with a minimum of 5 × 10^4^ cells per sample and all treatments were 2.5 μM. Mean RFU/cell is depicted by bars which correspond to the left axis, the percentage of nanoparticle positive cells is given by interleaved scatter plots that corresponds to the right axis. Images of endometrial cancer model spheroids following treatment (40× magnification). Images of Ishikawa (**b**) and Hec50 (**e**) control spheroids and spheroids treated with SAHA free, SAHA-NP and SAHA-NP-HA. Scale bars are 50 μm. Cytotoxicity of SAHA-NP delivery systems. Spheroid cytotoxicity assessment using Celltiter Glo 3D (*n* = 3) of SAHA Free Drug, SAHA-NP and SAHA-NP-HA at 2.5 μM for 48 h and 72 h in Ishikawa (**c**) and Hec50 (**f**). All data is normalized to the untreated control cells. Statistical significance is indicated on graphs: *p* ≤ 0.05 denoted by *, *p* ≤ 0.01 by ** and *p* ≤ 0.001 by ***.

**Figure 8:**
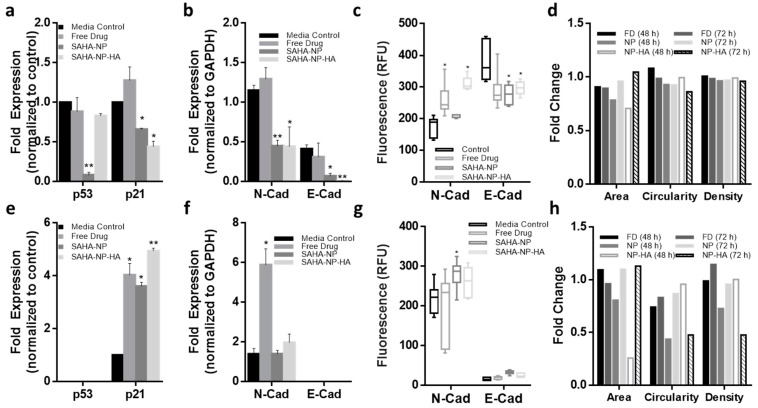
Effect of free and NP- encapsulated SAHA on p21 and p53 expression and molecular phenotype in 3D endometrial cancer models. Protein densitometry of media control (black) SAHA Free Drug (medium grey), SAHA-NP (dark grey) and SAHA-NP-HA (light grey), all treatments were 2.5 μM for 72 h. Protein analyses on cell cycle arrest markers p53 and p21 in Ishikawa (**a**) and Hec50 (**e**) and molecular phenotype markers E and N cadherin in Ishikawa (**b**) and Hec50 (**f**), * p53 and p21 results are average and standard error, from a minimum of three independent repeats, results are normalized to the untreated control cells and expressed as a ratio compared to housekeeping protein GAPDH density levels. Cadherin values are average and standard error, from a minimum of three independent repeats normalized to GAPDH protein density level only. Cadherin expression in spheroids Quantified immunofluorescence of E and N-cadherin in Ishikawa (**c**) and Hec50 (**g**) spheroids following treatments, relative fluorescence units (RFU) values obtained from ImageJ analysis of immunofluorescence images (*n* = 6), RFU per unit area. Spheroid morphology metrics ImageJ analysis of the spheroid images was conducted to ascertain change in area, circularity and density in Ishikawa (**d**) and Hec50 (**h**) following treatments, free drug 48 h (black), free drug 72 h (dark grey), SAHA-NP 48 h (medium grey), SAHA-NP 72 h (light grey), SAHA-NP-HA 48 h (white with grey outline) and SAHA-NP-HA 72 h (striped). All data was normalized to untreated control spheroids. Statistical significance is indicated on graphs: *p* ≤ 0.05 denoted by * and *p* ≤ 0.01 by **.

Cell seeding optimization determined that a seeding density of 4 × 10^3^ resulted in optimal spheroid growth for both Ishikawa and Hec50 (Figure S6), using image (Figure S7a,b) analysis and object area, circularity and density as broad indicators of successful establishment of the desired spheroid morphology and growth rate (Figure 7b,e and Figure 8d,h). Spheroid penetration and cellular uptake was investigated to further understand particle behavior. Spheroids were treated for 48 h with each NP formulation (loaded with PI), disaggregated and analyzed by flow cytometry. All NPs (including NP-S-S-) showed complete spheroid penetration with over 93% of cells being positive for PI. In Ishikawa (Figure 7a), a clear increase in intensity/cell is seen in NP-HA (mean of 2.9 × 10^5^) compared to all other treatments (NP, 2.2 × 10^5^; NP-S-S-, 2.1 × 10^5^). 3D NP uptake in Hec50 (Figure 7d) show consistency with the results observed in 2D. NP-HA mean intensity/cell was shown to be 1.8 × 10^5^ compared to 1.9 × 10^5^ in NP treatments (NP-S-S-, 1.4 × 10^5^). Interestingly, the interquartile range was shown to be higher for NP-HA than NP, conceivably due to the heterogenous expression of CD44 in Hec50 leading to a large spread of uptake throughout the population. Furthermore, both Ishikawa and Hec50 spheroids demonstrated a reduction in uptake when pre-treated with HA with mean intensities reduced by 0.5 × 10^5^ (Figure 7a) and 0.2 × 10^5^ (Figure 7d), respectively [39]. Across both cell lines NP and NP-HA have great spheroid penetration, as evidenced by over 99.5% of Ishikawa cells and over 98% of Hec50 cells being NP positive. HA saturation inhibited uptake of NP-HA by 1.8% in Ishikawa cells and 3.6% of Hec50 cells.

Ishikawa spheroids (Figure 7b and Figure S7a) were treated for 48 h with SAHA-NP and showed a 75% reduction in cellular viability compared to the untreated control (Figure 7c, *p* < 0.01), a 3.2-fold greater effect compared to free SAHA (*p* < 0.05). Similarly, Hec50 spheroids treated for 48 h with SAHA-NP showed a 40% reduction in cell viability when compared to the untreated control (*p* < 0.05), a 4-fold increase in cell death compared to the free drug (Figure 7f and Figure S7b). SAHA-NP-HA showed an increased cytotoxic effect in both Ishikawa and Hec50 spheroids. Hec50 cells, which express CD44 heterogeneously, but high levels in cells expressing the receptor, showed marked differences in cytotoxicity as well as spheroid morphology when treated with HA-NPs, indicating that the HA polymers cause loss of spheroid stability. Ishikawa 3D models exhibited a 75% reduction in cell viability following 48 h SAHA-NP treatment (*p* < 0.01) which increased to 86% with SAHA-NP-HA (*p* < 0.0001) (Figure 7c). Similarly, Hec50 viability was reduced by 40% after 48 h SAHA-NP treatment (*p* < 0.05) and again was increased, to 45%, with SAHA-NP-HA (*p* < 0.05; (Figure 7f). In addition, as the maximal drug release kinetics for SAHA-NP-HA occurred approximately 24 h after SAHA-NP, cytotoxicity of SAHA-NP-HA was also evaluated after 72 h and revealed that the effect of targeted delivery and release was further enhanced for both cell lines. (Figure 7a,c; *p* < 0.01).

As anticipated p53 expression was decreased in Ishikawa spheroids following treatments, reductions of 12%, 92% and 17% were observed in FD, SAHA-NP (*p* < 0.01) and SAHA-NP-HA, respectively. Interestingly p21 protein expression also decreased by 34% and 56% following treatment with SAHA-NP (*p* < 0.05) and SAHA-NP-HA (*p* < 0.05), respectively (FD treatment increased p21 expression by 27%) (Figure 8a and Figure S8a (original western blots can be found at Figures S12 and S13)). Studies have demonstrated that extrinsic apoptosis and death receptor functionality via TRAIL signaling can be re-instated following SAHA treatment [98,99]. In Hec50 spheroids, p21 expression was upregulated with all SAHA treatments (Figure 8e). In Ishikawa cells as N-cadherin levels increased with concomitant decrease in E-cadherin suggesting the cells undergo a phenotypic switch in response to SAHA in the 3D tumor model (Figure 8b and Figure S8b; *p* < 0.01). In Hec50 cells N-cadherin remained unaltered after 72 h treatment with SAHA-NP-HA (*p* > 0.05; Figure 8f).

Quantified immunofluorescence of N and E cadherin (Figure 6d,h) from z stack images of spheroids (Figure S8c–e) was carried out at depths of 6 and 13 μm. In Ishikawa (Figure 6h) mean N-cadherin RFU was increased from 175 to 259, 209 and 310 by free drug (*p* < 0.05), SAHA-NP and SAHA-NP-HA (*p* < 0.05), respectively. E-cadherin expression was downregulated from a mean RFU of 379 to 288, 276 and 297 by free drug, SAHA-NP (*p* < 0.05) and SAHA-NP-HA (*p* < 0.05), respectively. Immunofluorescence images of the untreated Ishikawa spheroids (Figure S8c,e) show a mosaic expression pattern of both cadherins (N-cadherin is shown in green, E-cadherin is shown in red), and after SAHA treatments there was a higher frequency of co-localization of cadherins (orange). This upregulation of N cadherin was also seen in Hec50 spheroids where mean RFU increased from 218 to 241, 280 and 261 following free drug, SAHA-NP (*p* < 0.05) and SAHA-NP-HA treatments, respectively.

Finally, assessment of spheroid morphology revealed that SAHA-NP treatment led to 15%, 20% and 4% reductions in Ishikawa spheroid area, circularity and density, respectively (Figure 8d and Figure S5a). SAHA-NP-HA treatment resulted in a further 15% reduction of spheroid area but had little additional effect on circularity and density. For Hec50 spheroids even greater reductions of 30%, 35% and 26% were observed for spheroid area, circularity and density, respectively (Figure 8h and Figure S7b). This reduction corresponded with the increased cytotoxicity and suggested increased spheroid penetration using HA functionalized particles may lead the observed increase in drug efficacy.

## 4. Conclusions

Advanced and aggressive endometrial cancers can be refectory to treatment via systemic administration of chemotherapies including cisplatin, carboplatin, doxorubicin and taxane. With the number of pre-menopausal woman diagnosed with endometrial cancer increasing, the development of new treatments remains imperative. The molecular pathogenesis of type I and II endometrial cancer has been linked to alterations in epigenetic processes including histone (de)acetylation, [15,16] therefore the development and effective and safe administration of compounds that can partially or fully restore the correct epigenetic phenotype including the re-expression of inactivated tumor suppressor genes and deactivation of activated oncogenes offer exciting prospects. Unfortunately, to date, the HDAC inhibitor SAHA has demonstrated little efficacy in endometrial cancer Phase II trials, [25] likely due to factors including poor stability in circulation and lack of accumulation at effective concentrations at the tumor sites due to poor tumor penetration and drug efflux [26].

Here we have developed a novel nanomedicine delivery system through coupling ‘regulator approved’ entities, namely Pluronic^®^ F-127 micelle nanoparticles, HA and SAHA. This approach is aimed at enabling the rapid development of efficacious SAHA delivery systems, rather than the fabrication of de novo particles, that would require extended regulatory approvals. Very importantly, we demonstrate that HA-SAHA-NP is most effective when used in 3D tumor models of both a type I and type II endometrial cancer which better represent tumors. The use of HA to target CD44 did not increase uptake efficiency in a 2D model, suggesting that targeting CD44 could an effective approach to enhancing penetration into solid tumors where this molecule is overexpressed. SAHA proved an effective agent in restoring p21 and p53 functionality through the expected mechanism of increased acetylation. Furthermore, our findings suggest that SAHA treatment can revert endometrial cancer cells to a less invasive phenotype through driving an EMT switch. Targeting epigenetic processes offers a novel approach to tackling the growing burden of endometrial cancer, and the targeting of simple, yet long-lived, nanoparticles containing these drugs could lead to a step change in the treatment of women inflicted with the malady.

## Figures and Tables

**Table 1:** Characterization of nanodelivery systems. Four different nanoparticle formulations were analyzed by size (DLS), polydispersity index (PDI; DLS), zeta potential (DLS), encapsulation efficiency (EE%) and drug release profile data. All data shown is average and standard deviation, from a minimum of three independent biological repeats. ** Statistically significant values compared to SAHA-NP.

Particle	Size Average (nm)	PDI	Zeta Potential (mV)	Encapsulation Efficiency (EE%)	Loading Capacity (LC%)	Drug Release Maxima (h)	Encapsulated Drug Released over 72 h	First Order Rate Constant
Empty NP	29.1 ± 2.1	0.243	0.120 ± 0.25	-	-	-	-	-
SAHA-NP	35.7 ± 3.6	0.354	−0.197 ± 0.15	18%	0.6%	2	28.06 µg	2.5 ± 0.2
SAHA-NP-S-S-	179.9 ± 15.0	0.229	−3.12 ± 0.61	42%	9.1%	0.5	44.61 µg	-
SAHA-NP-HA	251.6 ± 22.0	0.312	−14.23 ± 3.29	45% **	9.7% **	23.5	39.63 µg	0.3 ± 0.03

## Data Availability

Data are contained within this article and the Supplementary Material.

## Supplementary Materials

The following are available online at https://www.mdpi.com/article/10.3390/cancers13164032/s1, Figure S1: Functionalized material characterization. 1H NMR spectra in deuterated methanol of F127 pyridyl disulphide showing new peaks within the aromatic region (a). FT-IR spectroscopy of pluronic materials (b), showing weak peak at 663 cm^−1^ which can be attributed to C-S and C=N peak at 1706 cm^−1^ in the functionalized material, peaks not present in starting material. FT-IR of hyalronic acid materials (c), due to low degree of thiol functionalisation (7% per dissacharide repeat as deteremined by Ellman’s assay) and weak signal of sulfur peaks in IR, no clear peak can be attributed to any sulfur functionalisation group. Modified HA spectra shows a new peak at 1676 cm^−1^, which can be attributed to the C=O bond of the carbamate group formed following functionalisation. Figure S2: Immunofluorescence images used for CD44 expression quantification in endometrial cancer models. Images of Ishikawa and Hec50 monolayers used for quantification of CD44 expression. Figure S3: Nanoparticle Transmission Electron Microscopy (TEM) images. TEM analyses of SAHA-NP (a), SAHA-NP-S-S (b) and SAHA-NP-HA (c). Size analysis from TEM images processed using Ilastic and visualised as a box and whisker (5–95 percentile) plot (d). Figure S4: Propidium Iodide (PI)—NP uptake in 2D. Im-ages of Ishikawa and Hec50 monolayers taken at 40× (a) non-treated control (NT), 2.5 μM NP(PI), 2.5 μM HA-NP(PI) and 2.5 μM HA-NP(PI) following HA saturation (2h pre-treatment of 250μg per 1 × 10^5^ cells [1]). Images shown are merged channels where nuclei are stained by Hoescht (blue), membranes stained by WGA-Alexa 488 (green) and NP(PI) is shown as red, scale bar shown in Ishikawa non-treated image is 100 μm. Images were quantified (Figure 4a,d) using cellprofiler pipeline, where co-localisation of NP signal is positively correlated to a cell area by segmentation based on membrane staining. Z stack images taken using CLSM (b) Individual channels and merge showing presence of inter-cellular PI (b) PI localization, images are all channels merged. Figure S5: Effect of SAHA, SAHA-NP and SAHA-NP-HA drug systems on EMT markers E and N cadherin in 2D. Representative images of each channel and merges (a) and merged images used for quantitative analyses given in Figure 6d,h (b). Ratio of N:E cadherin (c). Figure S6: Images of Endometrial cancer model spheroids taken at 40× magnification. Time series images of Ishikawa (a) and Hec50 (b) spheroids formed using 2 × 10^3^, 4 × 10^3^ and 6 × 10^3^ cells in ultra-low adhesion (ULA) plates over a 72 h time period. Images include a 50 µm scale bar. Figure S7: Images of Endometrial cancer model spheroids following treatment taken at 40× magnification. Images of Ishikawa (a) and Hec50 (b) spheroids treated with SAHA free, SAHA-NP and SAHA-NP-HA, at both 48 and 72 h timepoints. Brightfield images of Ishikawa (a) and Hec50 (b) spheroids treated with SAHA free, SAHA-NP and SAHA-NP-HA, at both 48 and 72 h timepoints. Images were used for spheroid metric assessments. Figure S8: Effect of SAHA, SAHA-NP and SAHA-NP-HA drug systems on cell cycle progression and molecular phenotype from endometrial cancer cell line models in 3D. Representative example of Western Blots taken from Ishikawa (a) and Hec50 (b) spheroid protein isolates. Representative example of Western Blots used for densitometry work in Figure 8. Representative immunofluorescent images taken at 20× magnification (e) used for quantified IF expression of E and N cadherins in Figure 8. Examples of each channel and merges (c) and example spheroid area thresholding as determined by ImageJ (d). N:E cadherin ratio visualization (f). Figure S9: Immunoblots used in Figure 5 showing acetylation state of histone 3 following treatments. Quantitative analysis used data from all repeats. Figure S10: Immunoblots used in to generate Figure 6a—representative blots from protein analysis of 2D treatments. Bands used for figures have been high-lighted, quantitative analysis used data from all repeats. Figure S11: Immunoblots used in to generate Figure 6d—representative blots from protein analysis of 2D treatments. Bands used for figures have been highlighted, quantitative analysis used data from all repeats. Figure S12: Immunoblots used in to generate Figure S8a—representative blots from protein analysis of 2D treatments. Bands used for figures have been highlighted, quantitative analysis used data from all repeats. Figure S13: Immunoblots used in to generate Figure S8b—representative blots from protein analysis of 2D treatments. Bands used for figures have been highlighted, quantitative analysis used data from all repeats.

## References

[1] Lortet-Tieulent J., Ferlay J., Bray F., Jemal A. (2018). International patterns and trends in endometrial cancer incidence, 1978–2013. J. Natl. Cancer Inst..

[2] National Cancer Intelligence Network (NCIN) Outline of Uterine Cancer in the United Kingdom: Incidence, Mortality and Survival. http://www.ncin.org.uk/view?rid=2398#:~:text=In%20the%20UK%2C%20the%20incidence,3.2%20to%203.7%20per%20100%2C000.

[3] Bray F., Ferlay J., Soerjomataram I., Siegel R.L., Torre L.A., Jemal A. (2018). Global cancer statistics 2018: GLOBOCAN estimates of incidence and mortality worldwide for 36 cancers in 185 countries. CA Cancer J. Clin..

[4] Cancer Research UK (2021). Uterine Cancer Incidence.

[5] Morice P., Leary A., Creutzberg C., Abu-Rustum N., Darai E. (2016). Endometrial cancer. Lancet.

[6] Saso S., Chatterjee J., Georgiou E., Ditri A.M., Smith J.R., Ghaem-Maghami S. (2011). Endometrial cancer. BMJ.

[7] Frei K.A., Kinkel K. (2001). Staging endometrial cancer: Role of magnetic resonance imaging. J. Magn. Reson. Imaging.

[8] Doll A., Abal M., Rigau M., Monge M., Gonzalez M., Demajo S., Colás E., Llauradó M., Alazzouzi H., Planagumá J. (2008). Novel molecular profiles of endometrial cancer-new light through old windows. J. Steroid Biochem. Mol. Biol..

[9] Hoffman B.L., Schorge J.O., Schaffer J.I., Halvorson L.M., Bradshaw K.D., Cunningham F.G., Calver L.E. (2012). Chapter 15. Reproductive Endocrinology.

[10] Lodish H., Berk A., Zipursky S.L., Matsudaira P., Baltimore D., Darnell J., Freeman W.H. (2000). DNA Damage and Repair and Their Role in Carcinogenesis. Molecular Cell Biology.

[11] Vale C.L., Tierney J., Bull S.J., Symonds P.R. (2012). Chemotherapy for advanced, recurrent or metastatic endometrial carcinoma. Cochrane Database Syst. Rev..

[12] Risinger J.I., Maxwell G.L., Chandramouli G.V.R., Jazaeri A., Aprelikova O., Patterson T., Berchuck A., Barrett J.C. (2003). Microarray analysis reveals distinct gene expression profiles among different histologic types of endometrial cancer. Cancer Res..

[13] Nagashima M., Miwa N., Hirasawa H., Katagiri Y., Takamatsu K., Morita M. (2019). Genome-wide DNA methylation analysis in obese women predicts an epigenetic signature for future endometrial cancer. Sci. Rep..

[14] Tao M.H., Freudenheim J.L. (2010). DNA methylation in endometrial cancer. Epigenetics.

[15] Sakuragi N. (2013). Recent advances in research on epigenetic alterations and clinical significance of para-aortic lymphadenectomy in endometrial cancer: An introduction. Int. J. Clin. Oncol..

[16] Arafa M., Somja J., Dehan P., Kridelka F., Goffin F., Boniver J., Delvenne P. (2010). Current concepts in the pathology and epigenetics of endometrial carcinoma. Pathology.

[17] Bartosch C., Lopes J.M., Jerónimo C. (2017). Epigenetics in endometrial carcinogenesis—Part 1: DNA methylation. Epigenomics.

[18] Kandoth C., Schultz N., Cherniack A.D., Akbani R., Liu Y., Shen H., Robertson A.D., Pashtan I., Shen R., Cancer Genome Atlas Research Network (2013). Integrated genomic characterization of endometrial carcinoma. Nature.

[19] Lechner M., Boshoff C., Beck S. (2010). Cancer Epigenome. Adv. Genet..

[20] Ma X., Ma C.X., Wang J. (2014). Endometrial carcinogenesis and molecular signaling pathways. Am. J. Mol. Biol..

[21] Ren J., Zhang J., Cai H., Li Y., Zhang Y., Zhang X., Zhao D., Li Z., Ma H., Wang J. (2014). HDAC as a therapeutic target for treatment of endometrial cancers. Curr. Pharm. Des..

[22] Jiang S., Dowdy S.C., Meng X.W., Wang Z., Jones M.B., Podratz K.C., Jiang S.W. (2007). Histone deacetylase inhibitors induce apoptosis in both Type I and Type II endometrial cancer cells. Gynecol. Oncol..

[23] Takai N., Narahara H., Takai N., Narahara H. (2007). Human endometrial and ovarian cancer cells: Histone deacetylase inhibitors exhibit antiproliferative activity, potently induce cell cycle arrest, and stimulate apoptosis. Curr. Med. Chem..

[24] Fröhlich L.F., Mrakovcic M., Smole C., Zatloukal K. (2016). Molecular mechanism leading to SAHA-induced autophagy in tumor cells: Evidence for a p53-dependent pathway. Cancer Cell Int..

[25] Nervi C., De Marinis E., Codacci-Pisanelli G. (2015). Epigenetic treatment of solid tumours: A review of clinical trials. Clin. Epigenet..

[26] Kobayashi H., Watanabe R., Choyke P.L. (2014). Improving conventional enhanced permeability and retention (EPR) effects; What is the appropriate target?. Theranostics.

[27] Patra J.K., Das G., Fraceto L.F., Campos E.V.R., Rodriguez-Torres M.D.P., Acosta-Torres L.S., Diaz-Torres L.A., Grillo R., Swamy M.K., Sharma S. (2018). Nano based drug delivery systems: Recent developments and future prospects. J. Nanobiotechnol..

[28] Blanco E., Shen H., Ferrari M. (2015). Principles of nanoparticle design for overcoming biological barriers to drug delivery. Nat. Biotechnol..

[29] Jia L. (2005). Nanoparticle formulation increases oral bioavailability of poorly soluble drugs: Approaches, experimental evidences and theory. Curr. Nanosci..

[30] Ebeid K., Meng X., Thiel K.W., Do A.V., Geary S.M., Morris A.S., Pham E.L., Wongrakpanich A., Chhonker Y.S., Murry D.J. (2018). Synthetically lethal nanoparticles for treatment of endometrial cancer. Nat. Nanotechnol..

[31] Wilhelm S., Tavares A.J., Dai Q., Ohta S., Audet J., Dvorak H.F., Chan W.C.W. (2016). Analysis of nanoparticle delivery to tumours. Nat. Rev. Mater..

[32] Wang Y.-F., Liu L., Xue X., Liang X.-J. (2017). Nanoparticle-based drug delivery systems: What can they really do in vivo?. F1000Research.

[33] Safra T., Muggia F., Jeffers S., Tsao-Wei D.D., Groshen S., Lyass O., Henderson R., Berry G., Gabizon A. (2000). Pegylated liposomal doxorubicin (doxil): Reduced clinical cardiotoxicity in patients reaching or exceeding cumulative doses of 500 mg/m^2^. Ann. Oncol..

[34] Clift A.K., Drymousis P., Al-Nahhas A., Wasan H., Martin J., Holm S., Frilling A. (2015). Incidence of second primary malignancies in patients with neuroendocrine tumours. Neuroendocrinology.

[35] Minchinton A.I., Tannock I.F. (2006). Drug penetration in solid tumours. Nat. Rev. Cancer.

[36] Heldin C.H., Rubin K., Pietras K., Östman A. (2004). High interstitial fluid pressure—An obstacle in cancer therapy. Nat. Rev. Cancer.

[37] Rompicharla S.V.K., Trivedi P., Kumari P., Ghanta P., Ghosh B., Biswas S. (2017). Polymeric micelles of suberoylanilide hydroxamic acid to enhance the anticancer potential in vitro and in vivo. Nanomedicine.

[38] Xu J., Sun J., Wang P., Ma X., Li S. (2018). Pendant HDAC inhibitor SAHA derivatised polymer as a novel prodrug micellar carrier for anticancer drugs. J. Drug Target..

[39] Mattheolabakis G., Milane L., Singh A., Amiji M.M. (2015). Hyaluronic acid targeting of CD44 for cancer therapy: From receptor biology to nanomedicine. J. Drug Target..

[40] Tran T.H., Choi J.Y., Ramasamy T., Truong D.H., Nguyen C.N., Choi H.G., Yong C.S., Kim J.O. (2014). Hyaluronic acid-coated solid lipid nanoparticles for targeted delivery of vorinostat to CD44 overexpressing cancer cells. Carbohydr. Polym..

[41] Ghasemiyeh P., Mohammadi-Samani S. (2018). Solid lipid nanoparticles and nanostructured lipid carriers as novel drug delivery systems: Applications, advantages and disadvantages. Res. Pharm. Sci..

[42] Khatak S., Dureja H. (2015). Recent techniques and patents on solid lipid nanoparticles as novel carrier for drug delivery. Recent Pat. Nanotechnol..

[43] Jahangirian H., Lemraski E.G., Webster T.J., Rafiee-Moghaddam R., Abdollahi Y. (2017). A review of drug delivery systems based on nanotechnology and green chemistry: Green nanomedicine. Int. J. Nanomed..

[44] De Jong W.H., Borm P.J.A. (2008). Drug delivery and nanoparticles: Applications and hazards. Int. J. Nanomed..

[45] Jung H.H., Park K., Han D.K. (2010). Preparation of TGF-β1-conjugated biodegradable pluronic F127 hydrogel and its application with adipose-derived stem cells. J. Control Release.

[46] Akash M.S.H., Rehman K., Chen S. (2014). Pluronic F127-based thermosensitive gels for delivery of therapeutic proteins and peptides. Polym. Rev..

[47] Diniz I.M.A., Chen C., Xu X., Ansari S., Zadeh H.H., Marques M.M., Shi S., Moshaverinia A. (2015). Pluronic F-127 hydrogel as a promising scaffold for encapsulation of dental-derived mesenchymal stem cells. J. Mater. Sci. Mater. Med..

[48] Miyazaki S., Yokouchi C., Nakamura T., Hashiguchi N., Hou W.-M., Takada M. (1986). Pluronic F-127 gels as a novel vehicle for rectal administration of indomethacin. Chem. Pharm. Bull..

[49] Wang H., Williams G.R., Wu J., Wu J., Niu S., Xie X., Li S., Zhu L.M. (2019). Pluronic F127-based micelles for tumor-targeted bufalin delivery. Int. J. Pharm..

[50] Hong W., Shi H., Qiao M., Zhang Z., Yang W., Dong L., Xie F., Zhao C., Kang L. (2017). PH-sensitive micelles for the intracellular co-delivery of curcumin and Pluronic L61 unimers for synergistic reversal effect of multidrug resistance. Sci. Rep..

[51] Misra S., Heldin P., Hascall V.C., Karamanos N.K., Skandalis S.S., Markwald R.R., Ghatak S. (2011). Hyaluronan-CD44 interactions as potential targets for cancer therapy. FEBS J..

[52] Afify A.M., Craig S., Paulino A.F.G., Stern R. (2005). Expression of hyaluronic acid and its receptors, CD44s and CD44v6, in normal, hyperplastic, and neoplastic endometrium. Ann. Diagn. Pathol..

[53] Wojciechowski M., Krawczyk T., Śmigielski J., Malinowski A. (2015). CD44 expression in curettage and postoperative specimens of endometrial cancer. Arch. Gynecol. Obstet..

[54] Pulakkat S., Balaji S.A., Rangarajan A., Raichur A.M. (2016). Surface Engineered Protein Nanoparticles with Hyaluronic Acid Based Multilayers for Targeted Delivery of Anticancer Agents. ACS Appl. Mater. Interfaces.

[55] Choi K.Y., Min K.H., Na J.H., Choi K., Kim K., Park J.H., Kwon I.C., Jeong S.Y. (2009). Self-assembled hyaluronic acid nanoparticles as a potential drug carrier for cancer therapy: Synthesis, characterization, and in vivo biodistribution. J. Mater. Chem..

[56] Kim M., Hwang Y., Tae G. (2016). The enhanced anti-tissue adhesive effect of injectable pluronic-HA hydrogel by poly(γ-glutamic acid). Int. J. Biol. Macromol..

[57] Chen Y.Y., Wu H.C., Sun J.S., Dong G.C., Wang T.W. (2013). Injectable and thermoresponsive self-assembled nanocomposite hydrogel for long-term anticancer drug delivery. Langmuir.

[58] Kozak J., Wdowiak P., Maciejewski R., Torres A. (2018). A guide for endometrial cancer cell lines functional assays using the measurements of electronic impedance. Cytotechnology.

[59] Amaral R.L.F., Miranda M., Marcato P.D., Swiech K. (2017). Comparative analysis of 3D bladder tumor spheroids obtained by forced floating and hanging drop methods for drug screening. Front. Physiol..

[60] Costa E.C., Diogo D.M.D.M., Moreira A.F., Carvalho M.P., Correia I.J. (2018). Spheroids formation on non-adhesive surfaces by liquid overlay technique: Considerations and practical approaches. Biotechnol. J..

[61] Gurav D., Varghese O.P., Hamad O.A., Nilsson B., Hilborn J., Oommen O.P. (2016). Chondroitin sulfate coated gold nanoparticles: A new strategy to resolve multidrug resistance and thromboinflammation. Chem. Commun..

[62] Koivusalo L., Kauppila M., Samanta S., Parihar V.S., Ilmarinen T., Miettinen S., Oommen O.P., Skottman H. (2019). Tissue adhesive hyaluronic acid hydrogels for sutureless stem cell delivery and regeneration of corneal epithelium and stroma. Biomaterials.

[63] Caldwell K.M.E., Carlsson P.J.E., Li J.T. (1998). Coating of Hydrophobic Surfaces to Render them Protein Resistant while Permitting Covalent Attachment of Specific Ligands.

[64] Li J.-T., Carlsson J., Lin J.-N., Caldwell K.D. (1996). Chemical modification of surface active Poly(ethylene oxide)−Poly(propylene oxide) triblock copolymers. Bioconjug. Chem..

[65] Horcas I., Fernández R., Gómez-Rodríguez J.M., Colchero J., Gómez-Herrero J., Baro A.M. (2007). WSXM: A software for scanning probe microscopy and a tool for nanotechnology. Rev. Sci. Instrum..

[66] Kwak T.W., Kim D.H., Jeong Y.-I., Kang D.H. (2015). Antitumor activity of vorinostat-incorporated nanoparticles against human cholangiocarcinoma cells. J. Nanobiotechnol..

[67] Holmes K.A., Brown G.D., Carroll J.S. (2016). Chromatin immunoprecipitation-sequencing (ChLP-seq) for mapping of estrogen receptor-chromatin interactions in breast cancer. Methods in Molecular Biology.

[68] Hunter A.L., Narang N., Baxter M., Ray D.W., Poolman T.M. (2019). An improved method for quantitative ChIP studies of nuclear receptor function. J. Mol. Endocrinol..

[69] Idrees A., Chiono V., Ciardelli G., Shah S., Viebahn R., Zhang X., Salber J. (2018). Validation of in vitro assays in three-dimensional human dermal constructs. Int. J. Artif. Organs.

[70] Duellman S.J., Zhou W., Meisenheimer P., Vidugiris G., Cali J.J., Gautam P., Wennerberg K., Vidugiriene J. (2015). Bioluminescent, nonlytic, real-time cell viability assay and use in inhibitor screening. Assay Drug Dev. Technol..

[71] Kelly W.K., Marks P.A. (2005). Drug insight: Histone deacetylase inhibitors—Development of the new targeted anticancer agent suberoylanilide hydroxamic acid. Nat. Clin. Pr. Oncol..

[72] Komatsu N., Kawamata N., Takeuchi S., Yin D., Chien W., Miller C.W., Koeffler H.P. (2006). SAHA, a HDAC inhibitor, has profound anti-growth activity against non-small cell lung cancer cells. Oncol. Rep..

[73] Soderberg T. (2019). 15.7: Redox reactions of thiols and disulfides. Organic Chemistry with a Biological Emphasis Volume I.

[74] Edwardson J.M., Henderson R.M. (2004). Atomic force microscopy and drug discovery. Drug Discov. Today.

[75] Maver U., Velnar T., Gaberšček M., Planinšek O., Finšgar M. (2016). Recent progressive use of atomic force microscopy in biomedical applications. TrAC Trends Anal. Chem..

[76] (2011). Atomic Force Microscopy in Biomedical Research.

[77] Eaton P., Quaresma P., Soares C., Neves C., De Almeida M.P., Pereira E., West P. (2017). A direct comparison of experimental methods to measure dimensions of synthetic nanoparticles. Ultramicroscopy.

[78] Pisano S., Wang X., Garcia-Parra J., Gazze A., Edwards K., Feltracco V., Hu Y., He L., Gonzalez D., Francis L.W. (2020). Nanomicelles potentiate histone deacetylase inhibitor efficacy in vitro. Cancer Nanotechnol..

[79] Teulon J.M., Godon C., Chantalat L., Moriscot C., Cambedouzou J., Odorico M., Ravaux J., Podor R., Gerdil A., Habert A. (2019). On the operational aspects of measuring nanoparticle sizes. Nanomaterials.

[80] Gref R., Torchilin V., Minamitake Y., Peracchia M., Trubetskoy V., Langer R. (2006). Biodegradable long-circulating polymeric nanospheres. Science.

[81] Dinarvand R., Sepehri N., Manoochehri S., Rouhani H., Atyabi F. (2011). Polylactide-co-glycolide nanoparticles for controlled delivery of anticancer agents. Int. J. Nanomed..

[82] Gao P., Nie X., Zou M., Shi Y., Cheng G. (2011). Recent advances in materials for extended-release antibiotic delivery system. J. Antibiot..

[83] Stiefel P., Schmidt-Emrich S., Maniura-Weber K., Ren Q. (2015). Critical aspects of using bacterial cell viability assays with the fluorophores SYTO9 and propidium iodide. BMC Microbiol..

[84] Gomes-Alves A.G., Maia A.F., Cruz T., Castro H., Tomás A.M. (2018). Development of an automated image analysis protocol for quantification of intracellular forms of *Leishmania* spp.. PLoS ONE.

[85] Nykopp T.K., Rilla K., Tammi M.I., Tammi R.H., Sironen R., Hämäläinen K., Kosma V.M., Heinonen S., Anttila M. (2010). Hyaluronan synthases (HAS1-3) and hyaluronidases (HYAL1-2) in the accumulation of hyaluronan in endometrioid endometrial carcinoma. BMC Cancer.

[86] Nykopp T.K., Pasonen-Seppänen S., Tammi M.I., Tammi R.H., Kosma V.M., Anttila M., Sironen R. (2015). Decreased hyaluronidase 1 expression is associated with early disease recurrence in human endometrial cancer. Gynecol. Oncol..

[87] Paiva P., Van Damme M.P., Tellbach M., Jones R.L., Jobling T., Salamonsen L.A. (2005). Expression patterns of hyaluronan, hyaluronan synthases and hyaluronidases indicate a role for hyaluronan in the progression of endometrial cancer. Gynecol. Oncol..

[88] Li D., Marchenko N.D., Moll U.M. (2011). SAHA shows preferential cytotoxicity in mutant p53 cancer cells by destabilizing mutant p53 through inhibition of the HDAC6-Hsp90 chaperone axis. Cell Death Differ..

[89] Uchida H., Maruyama T., Nishikawa-Uchida S., Oda H., Miyazaki K., Yamasaki A., Yoshimura Y. (2012). Studies using an in vitro model show evidence of involvement of epithelial-mesenchymal transition of human endometrial epithelial cells in human embryo implantation. J. Biol. Chem..

[90] Tanaka Y., Terai Y., Kawaguchi H., Fujiwara S., Yoo S., Tsunetoh S., Takai M., Kanemura M., Tanabe A., Ohmichi M. (2013). Prognostic impact of EMT (epithelial-mesenchymal-transition)-related protein expression in endometrial cancer. Cancer Biol. Ther..

[91] Serrano-Gomez S.J., Maziveyi M., Alahari S.K. (2016). Regulation of epithelial-mesenchymal transition through epigenetic and post-translational modifications. Mol. Cancer.

[92] Lin H.Y., Chen C.S., Lin S.P., Weng J.R., Chen C.S. (2006). Targeting histone deacetylase in cancer therapy. Med. Res. Rev..

[93] Ropero S., Esteller M. (2007). The role of histone deacetylases (HDACs) in human cancer. Mol. Oncol..

[94] Lovitt C., Shelper T., Avery V. (2014). Advanced cell culture techniques for cancer drug discovery. Biology.

[95] Nirmalanandhan V.S., Duren A., Hendricks P., Vielhauer G., Sittampalam G.S. (2010). Activity of anticancer agents in a three-dimensional cell culture model. Assay Drug Dev. Technol..

[96] Lagies S., Schlimpert M., Neumann S., Wäldin A., Kammerer B., Borner C., Peintner L. (2020). Cells grown in three-dimensional spheroids mirror in vivo metabolic response of epithelial cells. Commun. Biol..

[97] Chitcholtan K., Asselin E., Parent S., Sykes P.H., Evans J.J. (2013). Differences in growth properties of endometrial cancer in three dimensional (3D) culture and 2D cell monolayer. Exp. Cell Res..

[98] Wu X., Yang N., Zhou W., Xu J., Chen J., Zheng F., Long Z., Yue C., Ai K., Liu L. (2014). Up-regulation of P21 inhibits TRAIL-mediated extrinsic apoptosis, contributing resistance to SAHA in acute myeloid leukemia cells. Cell. Physiol. Biochem..

[99] Zhou X., Liu Z., Wang H., Liu X., Zhou Z., Tang J., Liu X., Zheng M., Shen Y. (2019). SAHA (vorinostat) facilitates functional polymer-based gene transfection via upregulation of ROS and synergizes with TRAIL gene delivery for cancer therapy. J. Drug Target..

