# Supplementary material for: Hyaluronic Acid-Functionalized Nanomicelles Enhance SAHA Efficacy in 3D Endometrial Cancer Models
Source: Cancers (Basel). 2021 Aug 10;13(16):4032. doi: 10.3390/cancers13164032 (PMC8394402; doi:10.3390/cancers13164032)
Supplement: Supplementary file 1 [file cancers-13-04032-s001.zip › cancers-1311315-supplementary.pdf]

Supplementary materials: HA-Functionalized Nanomicelles Enhance SAHA Efficacy in 3D Endometrial Cancer Models

Kadie Edwards, Seydou Yao, Simone Pisano, Veronica Feltracco, Katja Brusehafer, Sumanta Samanta, Oommen P. Oommen, Andrea Gazze, Roberta Paravati, Holly Maddison, Chao Li, Deyarina Gonzalez, R. Steven Conlan and Lewis Francis

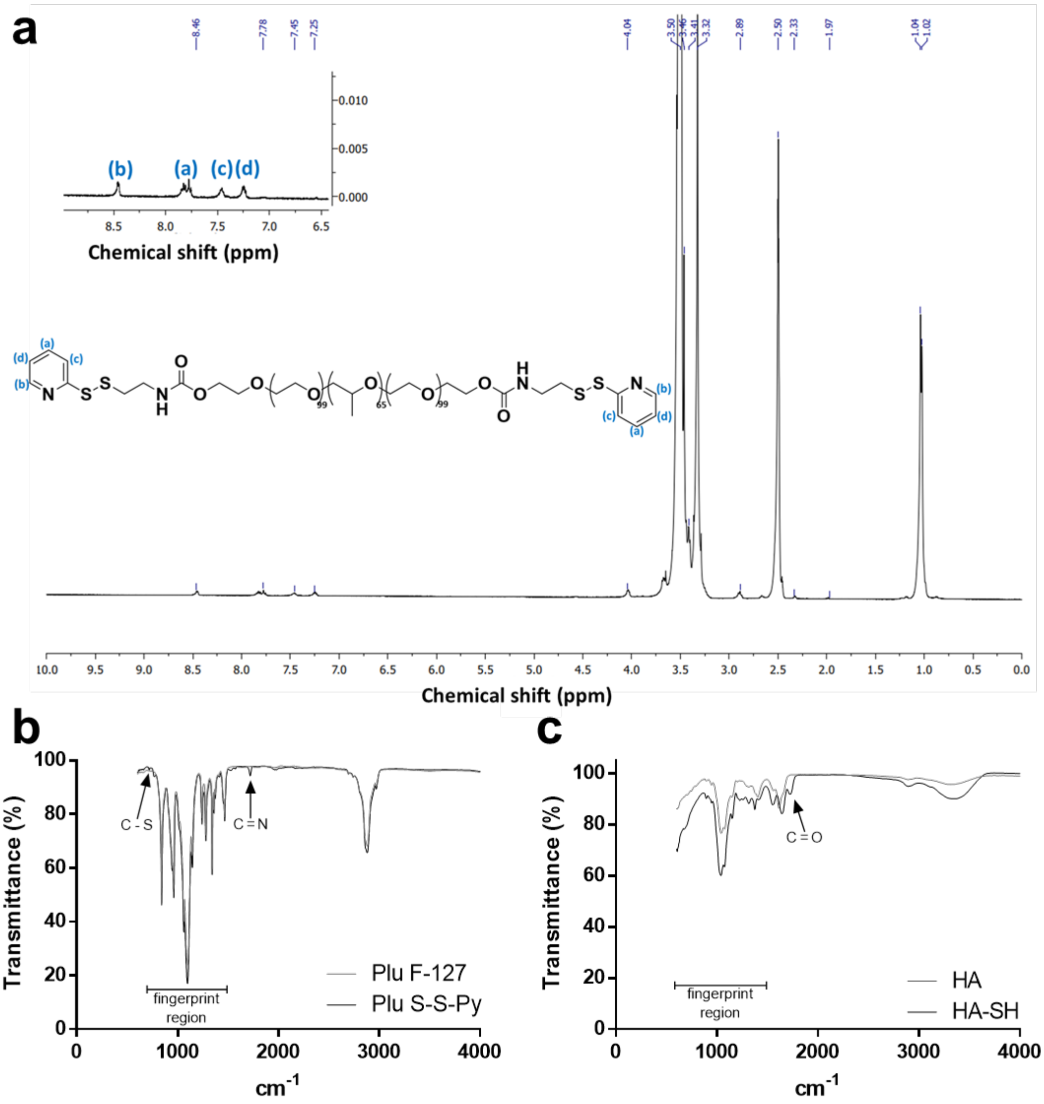

Figure S1. Functionalized material characterization. ^{1}H NMR spectra in deuterated methanol of F127 pyridyl disulphide showing new peaks within the aromatic region **(a)**. FT-IR spectroscopy of pluronic materials **(b)**, showing weak peak at 663cm^{-1} which can be attributed to C-S and C=N peak at 1706cm^{-1} in the functionalized material, peaks not present in starting material. FT-IR of hyalronic acid materials **(c)**, due to low degree of thiol functionalisation (7% per dissacharide repeat as deteremined by Ellman's assay) and weak signal of sulfur peaks in IR, no clear peak can be attributed to any sulfur functionalisation group. Modified HA spectra shows a new peak at 1676cm^{-1}, which can be attributed to the C=O bond of the carbamate group formed following functionalisation.

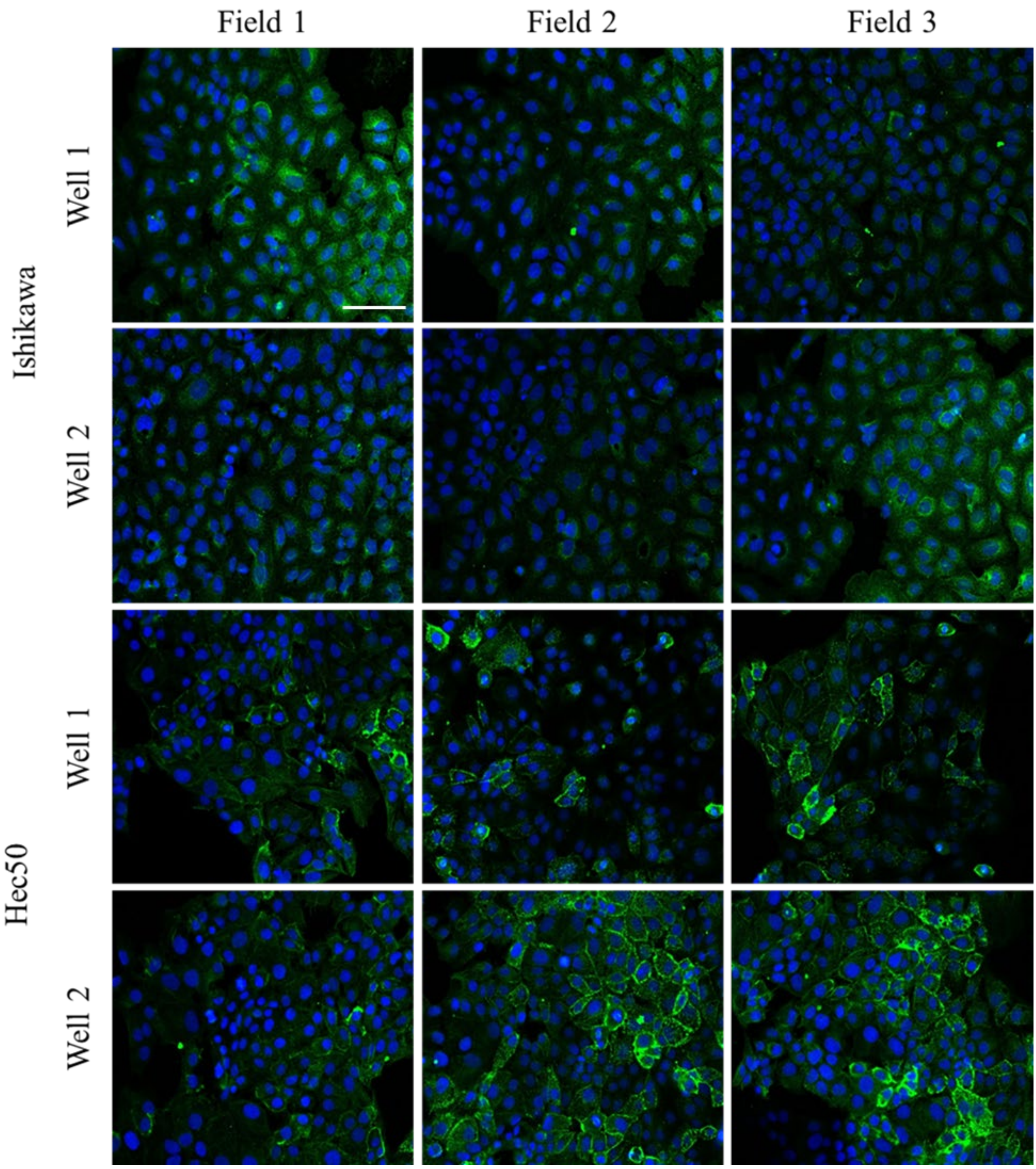

Figure S2. Immunofluorescence images used for CD44 expression quantification in endometrial cancer models. Images of Ishikawa and Hec50 monolayers used for quantification of CD44 expression. Scale bar is 100 µm.

Intermediate NP characterisation (NP-S-S)

Following drug incorporation, SAHA-NP-S-S displayed a hydrodynamic size of 179.9 ± 15.0 nm (**Table 1**) and a zeta potential of -3.12 ± 0.61 mV ($p < 0.05$). This drop in zeta potential from -0.197 ± 0.15 mV seen in the base formulation indicates successful micelle fabrication from the modified polymer and the marked difference compared to the functionalised particle (-14.23 ± 3.29 mV) clearly demonstrates successful HA coating. SAHA encapsulation was measured to be 45%, this number drops to 42% post-HA addition.

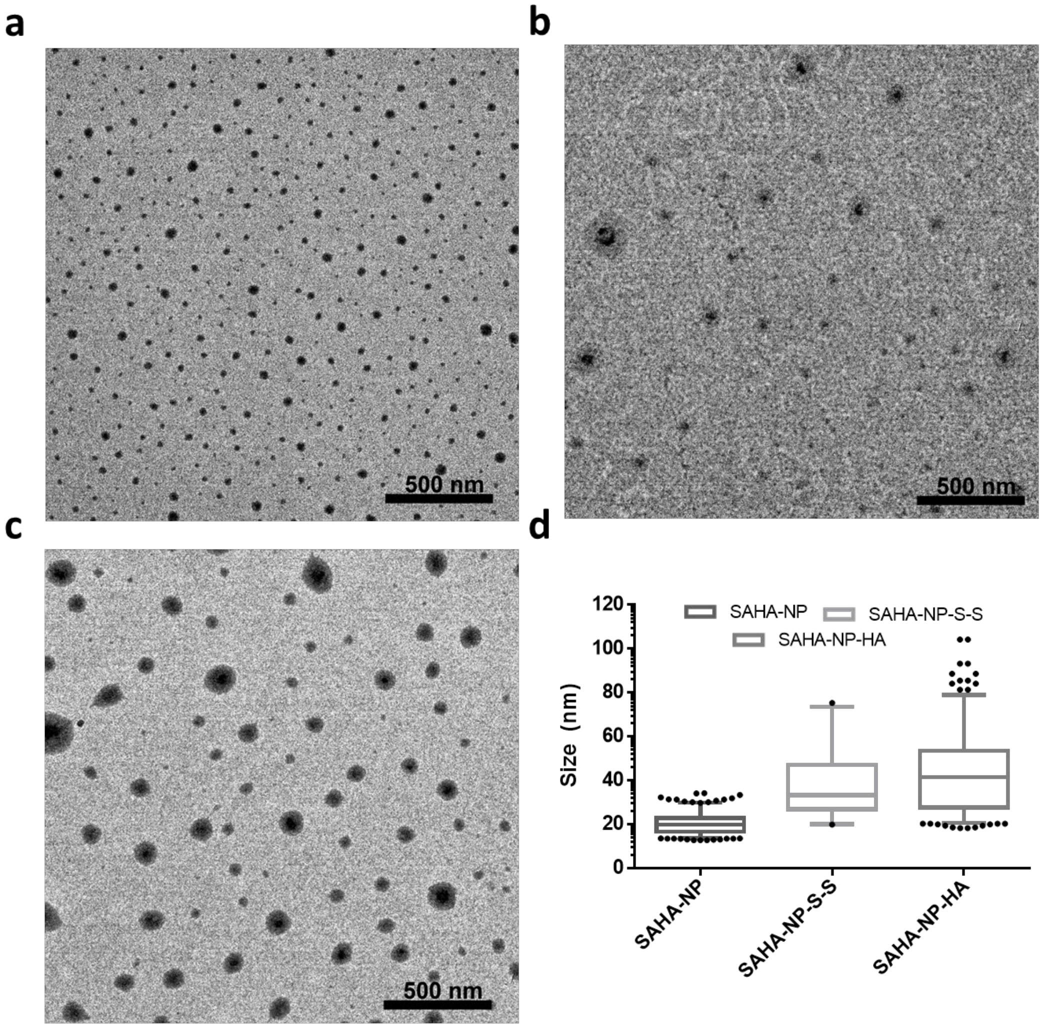

Figure S3. Nanoparticle Transmission Electron Microscopy (TEM) images. TEM analyses of SAHA-NP (**a**), SAHA-NP-S-S (**b**) and SAHA-NP-HA (**c**). Size analysis from TEM images processed using Ilastic and visualised as a box and whisker (5-95 percentile) plot (**d**).

Transmission Electron Microscopy

200-mesh formvar and carbon-coated copper grids (Agar Scientific) were incubated for 30 min with the samples, which were previously fixed for 30 min in 0.1% paraformaldehyde (Fisher Scientific) in PBS. Grids were washed in ultrapure water and stained with 2% phosphotungstic acid (Fisher Scientific) in water, pH 7. Excess solution was removed with filter paper and grids were allowed to air-dry. Grids were imaged on a JEM-1400Flash Transmission Electron Microscope at 80 kV. Images were pre-processed using Ilastic and ImageJ was used to identify and calculate dimensions of micelles. Data were plotted using GraphPad.

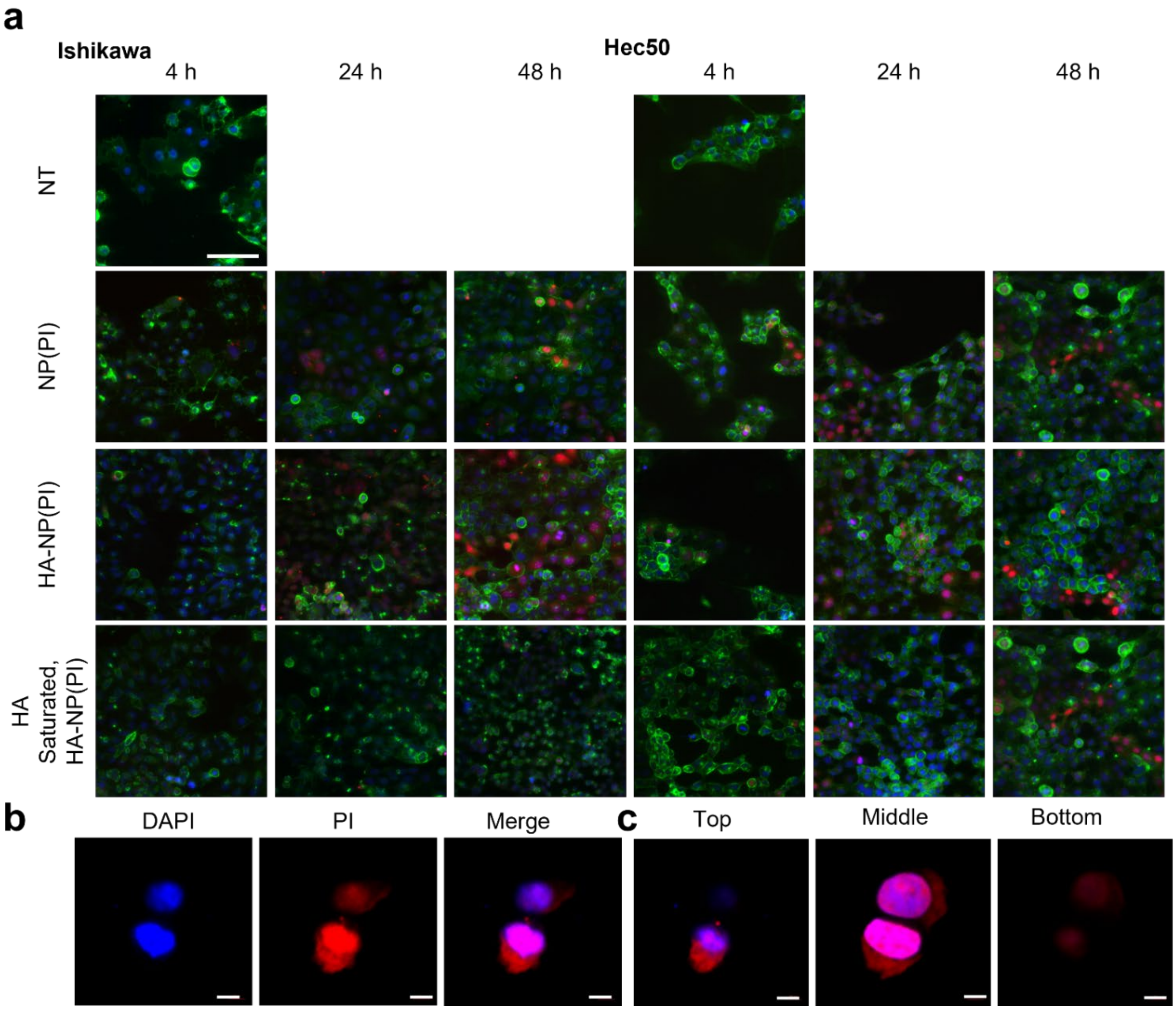

Figure S4. Propidium Iodide (PI) - NP uptake in 2D. Images of Ishikawa and Hec50 monolayers taken at 40× **(a)** non-treated control (NT), 2.5μM NP(PI), 2.5μM HA-NP(PI) and 2.5μM HA-NP(PI) following HA saturation (2 h pre-treatment of 250 μg per 1×10^5 cells [1]). Images shown are merged channels where nuclei are stained by Hoescht (blue), membranes stained by WGA-Alexa 488 (green) and NP(PI) is shown as red, scale bar shown in Ishikawa non-treated image is 100 μm. Images were quantified (**Figure 4a,d)** using cellprofiler pipeline, where co-localisation of NP signal is positively correlated to a cell area by segmentation based on membrane staining. Z stack images taken using CLSM **(b)** Individual channels and merge showing presence of intercellular PI **(b)** PI localization, images are all channels merged.

Immunofluorescence analysis

Cells (2×10^4 cells per well) were grown in 8 wells imaging chamber with microscope slides (Labtek) and analysed using a Zeiss LSM 710 confocal microscope or IN CELL analyser 2200 (GE Healthcare, UK). Propidium iodide (Sigma-Aldrich) free and encapsulated in the micelle were used as treatments at a concentration of 0.4 mg/mL. After 48 h incubation at 37 °C, 70% methanol were added for 5 mins to a well containing only cells and used as a positive control. After treatment, the wells were washed with PBS and fixed with 4% PFA (paraformaldehyde) for 10 min at 4 °C. All fixative was removed, and the cells stained with Hoescht (Life Technologies; 0.5 μg/ml) for 10 min before analysis. Image data was recorded in two emission spectra channels; DAPI nuclear (Channel 1 ⋌470 nm) and Texas Red for propidium iodide (Channel 2 ⋌620nm). A total of 3×10^4cells (30 images of approx. 1×10^3 cells) were obtained using a 20× objective, across a random distribution of the fields, excluding regions of the centre or edge of the well. Image analysis was performed using IN Cell Analyzer Workstation 3.5 software (IN Cell Investigator, GE Healthcare, UK). Briefly, an object segmentation feature masked the nuclei by segmenting on the basis of intensity in the DAPI channel. The high contrast in fluorescence output between the nuclear material and background facilitated efficient separation of nuclei against background. The masked bitmap was eroded in order to separate clumped nuclei prevalent in the monolayer and initial

segmentation repeated as above, to separate nuclei in proximity as individual objects using the eroded mask from the previous step. A 10 µm collar drawn around the nuclear mask isolated the cytoplasmic region of each cell. Fluorescence output in absolute grey levels was recorded pixel by pixel across the cell area mask and each cell was assigned a value for the propidium iodide expression based upon the average pixel fluorescence in the area. The software allows for calculation of total cellular fluorescence and the frequency of appearance was plotted for each treatment.

Confocal laser microscopy analysis

Cell samples were prepared and treated as above. The plate analysed using a Zeiss LSM 710 confocal microscope. Zen lite 2012 imaging software (Carl Zeiss Ltd, UK) was used for imaging analysis. Confocal quantification was performed using ImageJ software.

In vitro stability and uptake of NP- F-127 was assessed using propidium iodide uptake assays. Propidium iodide was encapsulated to the F-127 micelle during fabrication, and these fluorescent micelles used to quantify cellular internalisation. Propidium iodide cannot penetrate intact membranes without the F-127 carrier therefore the fluorescence is only observed following NPs were fabricated as described, substituting SAHA for propidium iodide to enable NP tracking, cellular internalisation and uptake.

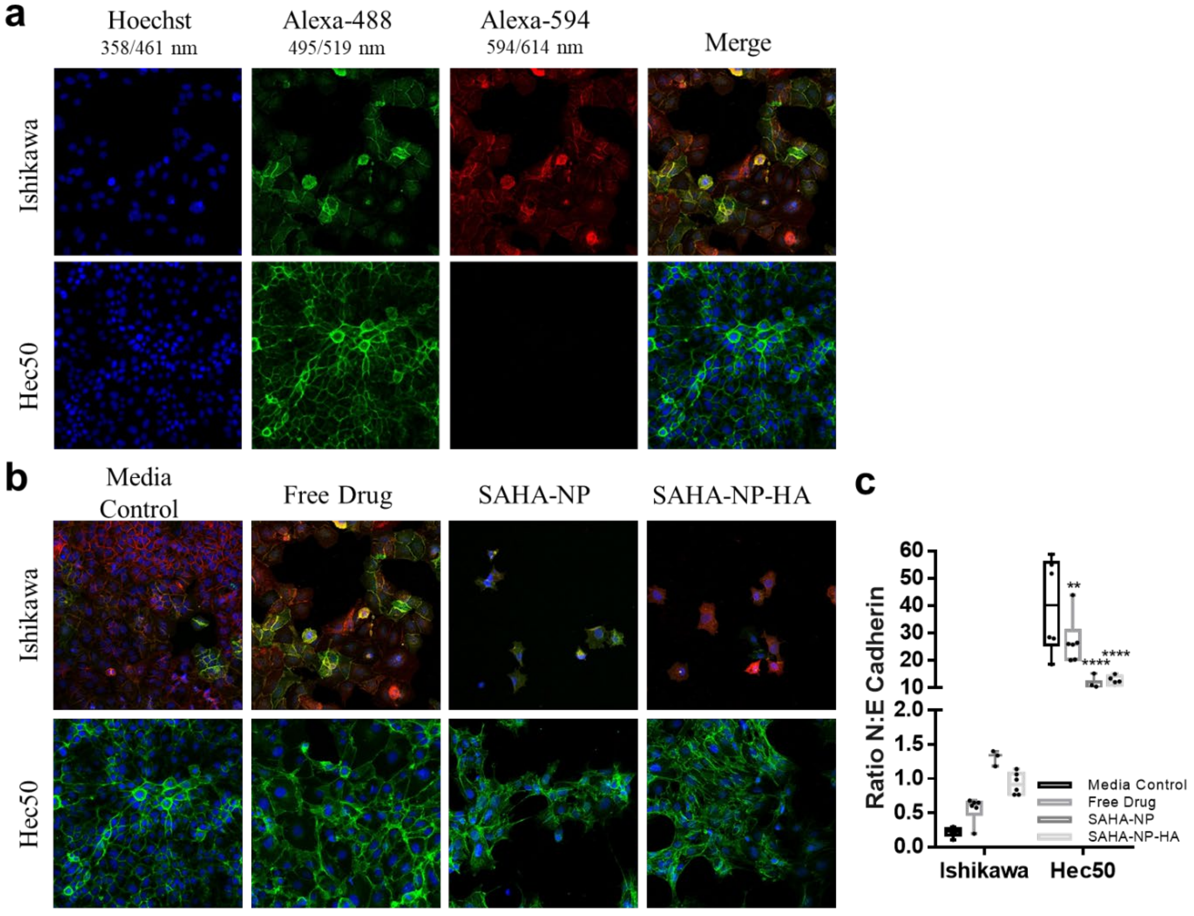

Figure S5. Effect of SAHA, SAHA-NP and SAHA-NP-HA drug systems on EMT markers E and N cadherin in 2D. Representative images of each channel and merges (**a**) and merged images used for quantitative analyses given in figure 6d and h (**b**). Ratio of N:E cadherin (**c**).

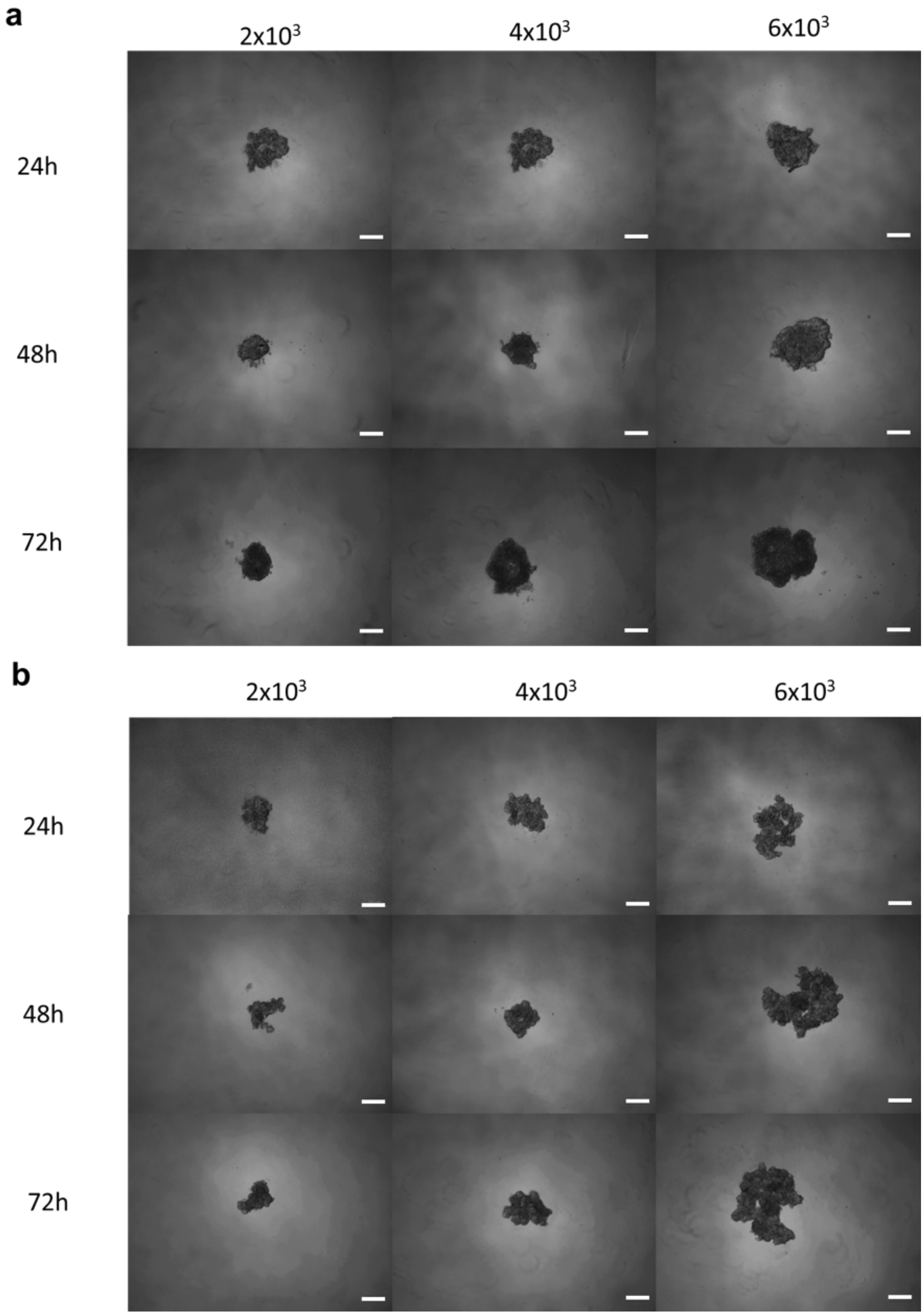

Figure S6. Images of Endometrial cancer model spheroids taken at 40× magnification. Time series images of Ishikawa (a) and Hec50 (b) spheroids formed using 2×10^3, 4×10^3 and 6×10^3 cells in ultra-low adhesion (ULA) plates over a 72 h time period. Images include a 50μm scale bar.

Ishikawa and hec50 Spheroid culture optimisation.

Spheroid optimization was conducted to determine an appropriate seeding density that would allow spheroid formation with the desired shape and mass (Figure S5). Spheroids grown from 2×10^3 gave poor initial association, did not show any change in shape or size between 24 h and 72 h. Hec50 spheroids grown from 6×10^3cells resembled cellular agglomerates and did not follow the same radial growth or morphology as that seen in Ishikawa. As both these densities gave unfavourable spheroids, 4×10^3 cells were used for all spheroids.

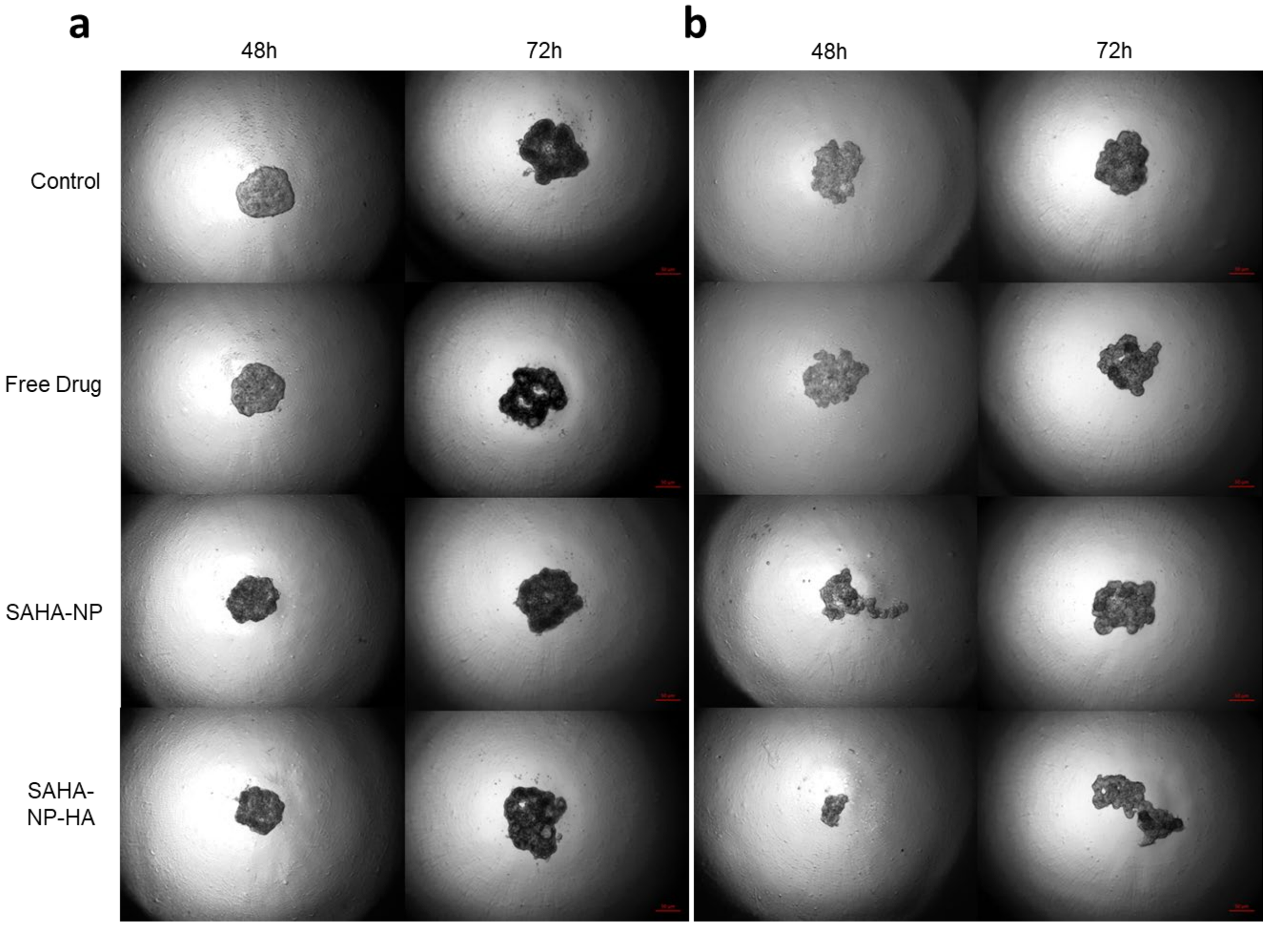

Figure S7. Images of Endometrial cancer model spheroids following treatment taken at 40X magnification. Images of Ishikawa (**a**) and Hec50 (**b**) spheroids treated with SAHA free, SAHA-NP and SAHA-NP-HA, at both 48 and 72 h timepoints. Brightfield images of Ishikawa (**a**) and Hec50 (**b**) spheroids treated with SAHA free, SAHA-NP and SAHA-NP-HA, at both 48 and 72 h timepoints. Images were used for spheroid metric assessments.

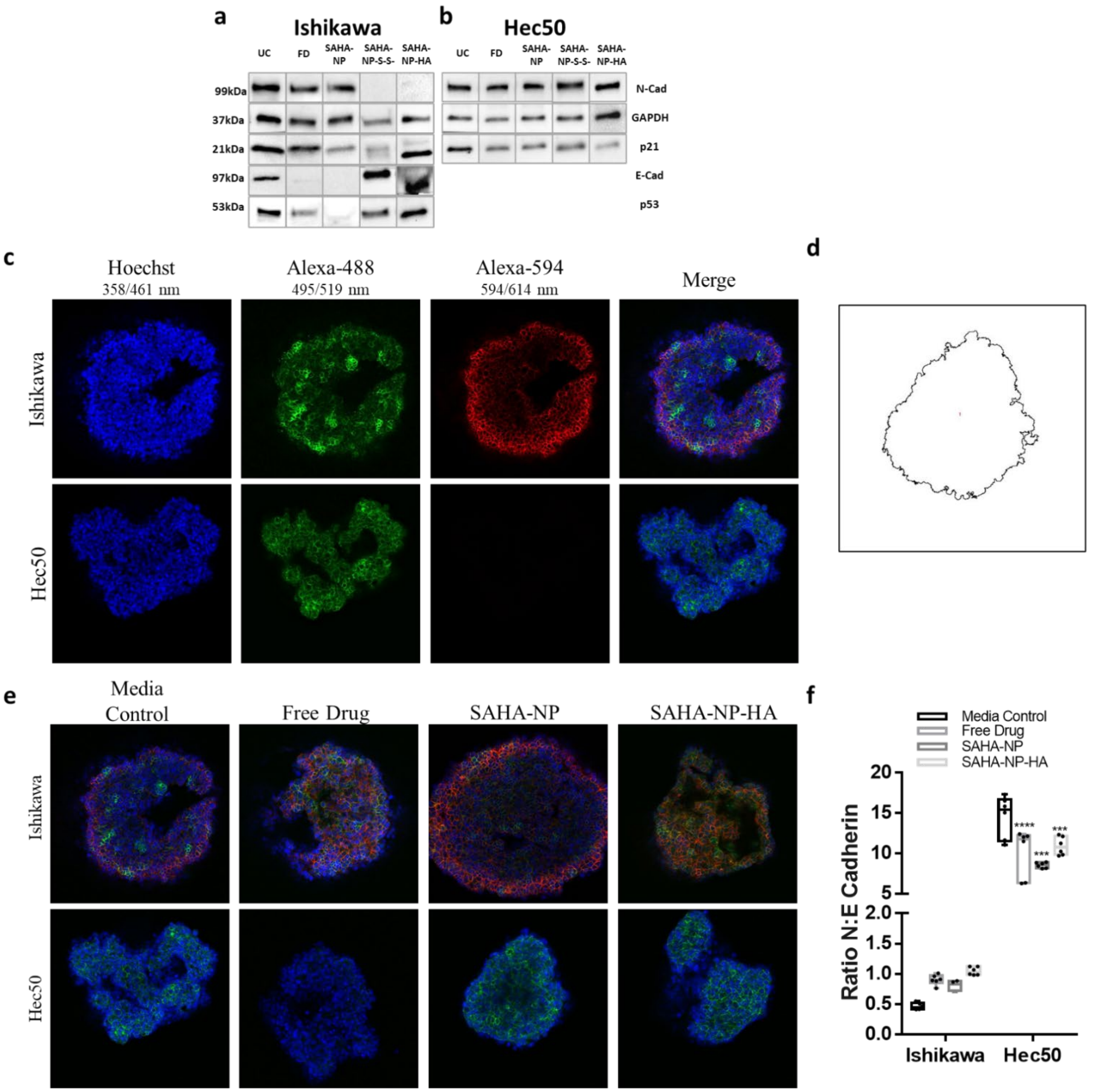

Figure S8. Effect of SAHA, SAHA-NP and SAHA-NP-HA drug systems on cell cycle progression and molecular phenotype from endometrial cancer cell line models in 3D. Representative example of Western Blots taken from Ishikawa (**a**) and Hec50 (**b**) spheroid protein isolates. Representative example of Western Blots used for densitometry work in Figure 8. Representative immunofluorescent images taken at 20× magnification (**e**) used for quantified IF expression of E and N cadherins in figure 8. Examples of each channel and merges (**c**) and example spheroid area thresholding as determined by ImageJ (**d**). N:E cadherin ratio visualization (**f**).

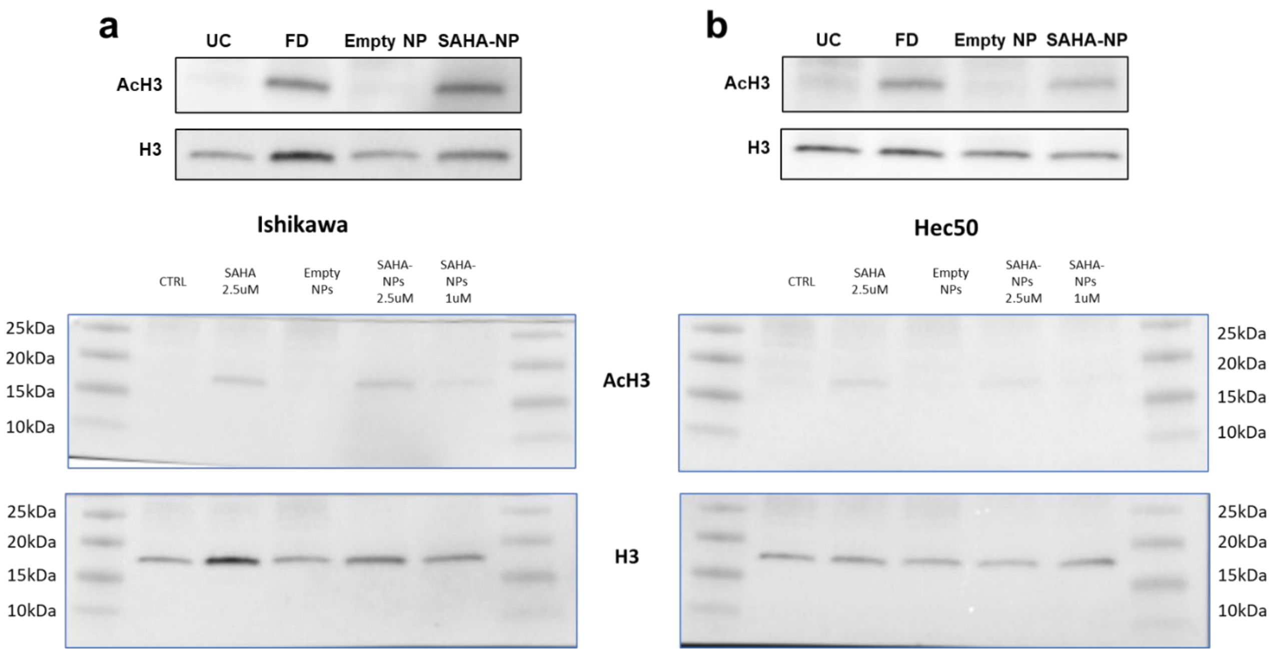

Figure S9. Immunoblots used in figure 5 showing acetylation state of histone 3 following treatments. Quantitative analysis used data from all repeats.

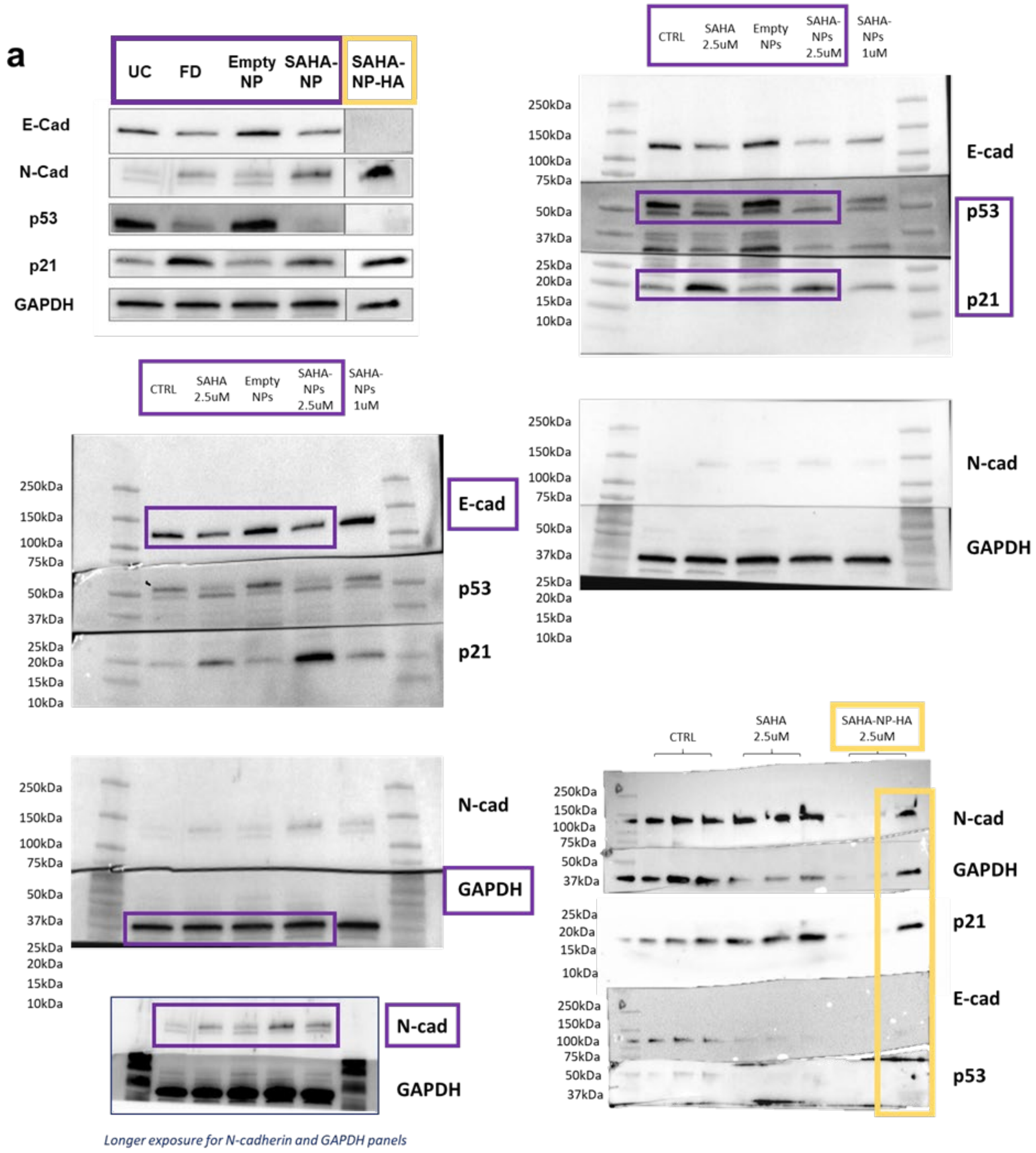

Figure S10. Immunoblots used in to generate Figure 6a – representative blots from protein analysis of 2D treatments. Bands used for figures have been highlighted, quantitative analysis used data from all repeats.

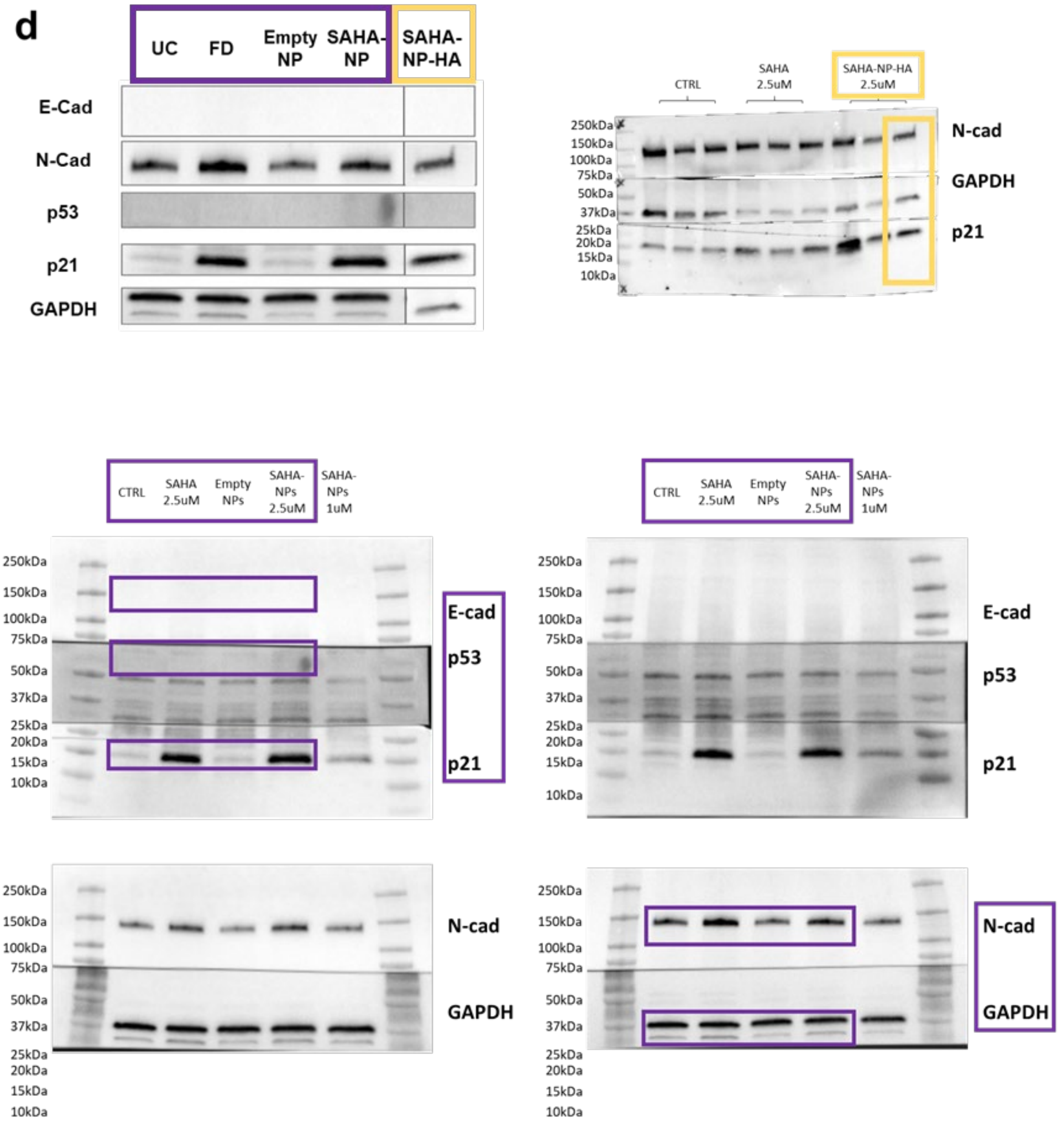

Figure S11. Immunoblots used in to generate Figure 6d – representative blots from protein analysis of 2D treatments. Bands used for figures have been highlighted, quantitative analysis used data from all repeats.

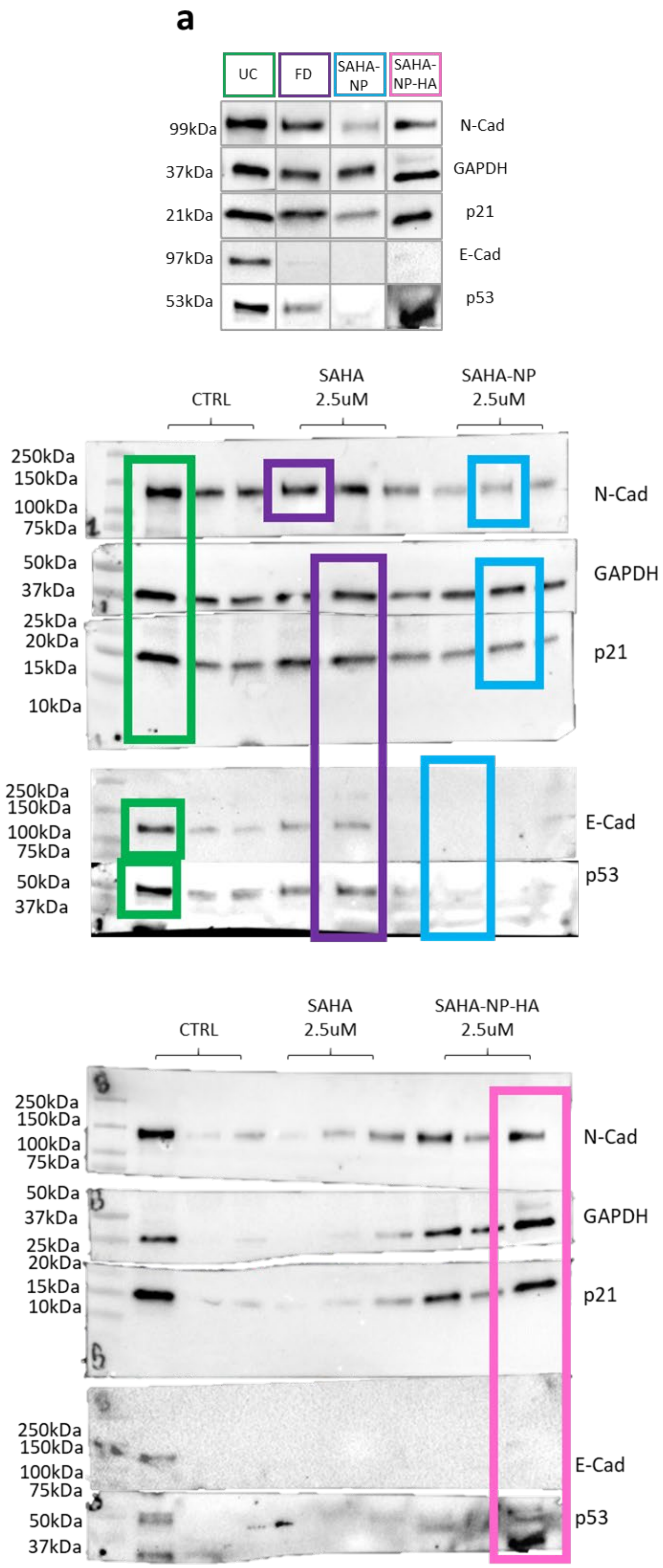

Figure S12. Immunoblots used in to generate Figure S8a—representative blots from protein analysis of 2D treatments. Bands used for figures have been highlighted, quantitative analysis used data from all repeats.

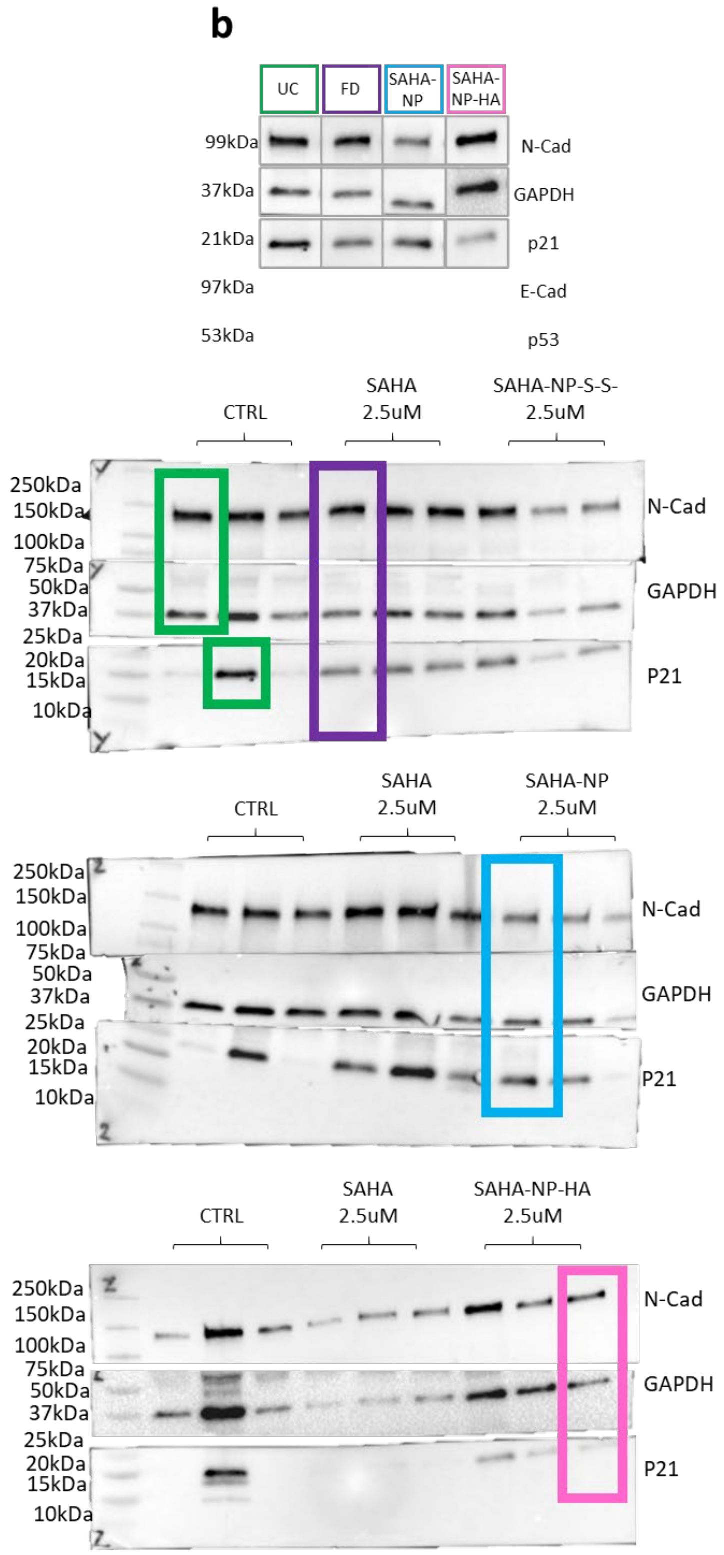

Figure S13. Immunoblots used in to generate Figure S8b—representative blots from protein analysis of 2D treatments. Bands used for figures have been highlighted, quantitative analysis used data from all repeats.
